# Optimization of metabolic oligosaccharide engineering with Ac_4_GalNAlk and Ac_4_GlcNAlk by an engineered pyrophosphorylase

**DOI:** 10.1021/acschembio.1c00034

**Published:** 2021-04-09

**Authors:** Anna Cioce, Ganka Bineva-Todd, Anthony J. Agbay, Junwon Choi, Thomas M. Wood, Marjoke F. Debets, William M. Browne, Holly L. Douglas, Chloe Roustan, Omur Y. Tastan, Svend Kjaer, Jacob T. Bush, Carolyn R. Bertozzi, Benjamin Schumann

**Affiliations:** a Department of Chemistry, Imperial College London, 80 Wood Lane, W12 0BZ, London, United Kingdom; b The Chemical Glycobiology Laboratory, The Francis Crick Institute, 1 Midland Rd, NW1 1AT London, United Kingdom; c Department of Chemistry, Stanford University, Stanford, CA 94305, USA; d Mycobacterial Metabolism and Antibiotic Research Laboratory, The Francis Crick Institute, 1 Midland Rd, NW1 1AT London, United Kingdom; e Structural Biology Science Technology Platform, The Francis Crick Institute, NW1 1AT London, United Kingdom; f GlaxoSmithKline, Gunnels Wood Road, Stevenage, Hertfordshire, SG1 2NY U; g Howard Hughes Medical Institute, 380 Roth Way, Stanford, CA 94305, USA

## Abstract

Metabolic oligosaccharide engineering (MOE) has fundamentally contributed to our understanding of protein glycosylation. Efficient MOE reagents are activated into nucleotide-sugars by cellular biosynthetic machineries, introduced into glycoproteins and traceable by bioorthogonal chemistry. Despite their widespread use, the metabolic fate of many MOE reagents is only beginning to be mapped. While metabolic interconnectivity can affect probe specificity, poor uptake by biosynthetic salvage pathways may impact probe sensitivity and trigger side reactions. Here, we use metabolic engineering to turn the weak alkyne-tagged MOE reagents Ac_4_GalNAlk and Ac_4_GlcNAlk into efficient chemical tools to probe protein glycosylation. We find that bypassing a metabolic bottleneck with an engineered version of the pyrophosphorylase AGX1 boosts nucleotide-sugar biosynthesis and increases bioorthogonal cell surface labeling by up to two orders of magnitude. Comparison with known azide-tagged MOE reagents reveals major differences in glycoprotein labeling, substantially expanding the toolbox of chemical glycobiology.

## Introduction

**Fig. 1:**
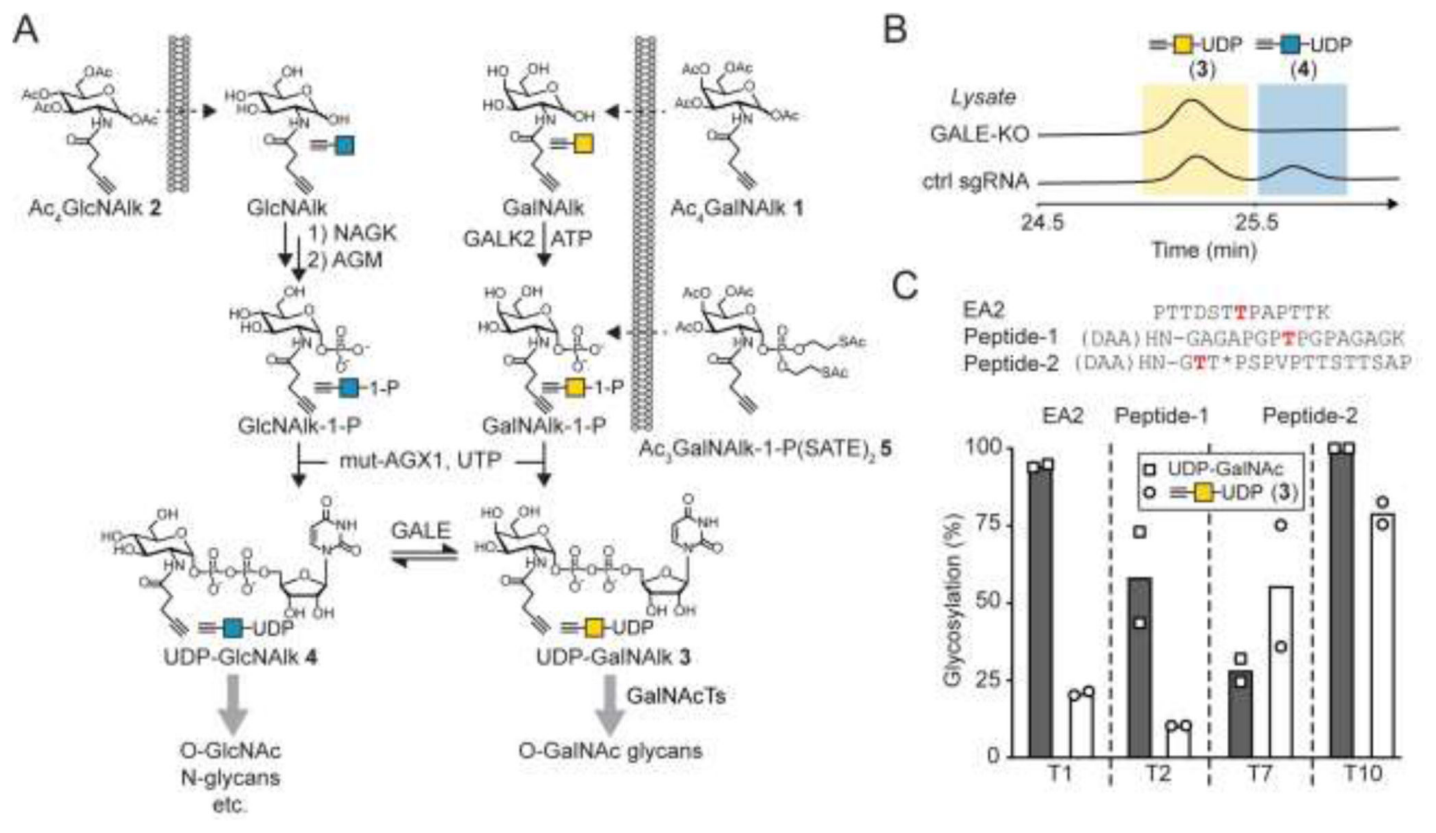
The metabolic fate of GalNAlk and GlcNAlk. *A*, biosynthesis of UDP-GalNAlk **3** and UDP-GlcNAlk **4** from caged precursors using salvage pathways. Dashed arrows indicate diffusion across membranes and (thioesterases). *B*, *in vitro* epimerization of UDP-GalNAlk **3** (yellow) to UDP-GlcNAlk **4** (blue) using a GALE-containing cell lysate, or a GALE-KO lysate as a control as assessed by HPAEC. The reaction was also performed using purified GALE, and retention times were compared to standards (Fig. S1). *C*, *in vitro* glycosylation with purified GalNAc-Ts of synthetic peptides using UDP-GalNAlk **3** or UDP-GalNAc as substrates. Red amino acids are new glycosylation sites. T* denotes a-D-GalNAc-Thr Data are individual measurements of biological duplicates and means. The reactions using UDP-GalNAc as a substrate have been used previously.^11^

Protein glycosylation is an important modulator of biological processes. Chemical MOE reagents have developed into important alternatives to protein-based binding reagents to profile the roles of glycans in cellular processes.^1–4^ Monosaccharides with chemical modifications can be fed to living cells as hydrophobic caged analogs that cross the plasma membrane, are deprotected by esterases, metabolically activated and introduced into the glycome by the activity of glycosyltransferases (GTs).^1,4–6^ Modifications such as azides or alkynes can be probed by bioorthogonal ligation using Cu(I)-assisted azide-alkyne cycloaddition (CuAAC) to allow for visualization and characterization of glycoconjugates.^2,4,7,8^ While it is generally accepted that small chemical perturbations are compatible with metabolic activation, the actual fate and turnover efficiency of modified monosaccharides is only beginning to be understood. Key to use by GTs is the biosynthesis of modified nucleotide-sugars, such as derivatives of uracil diphosphate (UDP)-activated *N*-acetylgalactosamine (GalNAc) and *N*-acetylglucosamine (GlcNAc) (Fig. 1A). The salvage pathway of GalNAc derivatives features the kinase GALK2 and the pyrophosphorylases AGX1/2, while GlcNAc derivatives have to be activated by the kinase NAGK, the mutase AGM as well as AGX1/2.^9,10^ In the cytosol of mammalian cells, derivatives of UDP-GalNAc and UDP-GlcNAc can be interconverted by the UDP-GalNAc/GlcNAc 4’-epimerase GALE, which interconnects both nucleotide-sugar pools. Epimerization substantially decreases the glycan specificity while enhancing the labeling efficiency of certain MOE reagents and can be suppressed by careful choice of the chemical modification.^10–12^ Once biosynthesized, derivatives of UDP-GalNAc and UDP-GlcNAc can be used as substrates by cellular GTs, including the large polypeptide GalNAc transferase (GalNAc-T) family in the secretory pathway and a myriad of GlcNAc transferases in several cellular compartments.

Recent years have seen increasing evaluation of the metabolic fate of MOE reagents. Although the enzymes of GalNAc and GlcNAc salvage pathways generally display reduced efficiency towards modifications on the acetamide side chain, the relatively small azide group is accepted as part of reliable MOE reagents.^10,12–15^ In contrast, large modifications prevent enzymatic activation of GalNAc and GlcNAc analogs.^11,16,17^ Yu et al. thus developed an engineered version of AGX1 (mutant F383G) to increase substrate promiscuity and biosynthesize UDP-GlcNAc analogs from the corresponding GlcNAc-1-phosphate analogs that can be delivered through caged precursors.^16^ We have used the similar F383A mutant, herein termed mut-AGX1, to biosynthesize UDP-GalNAc analogs that would normally not be made in the living cell.^4,11,17^ Somewhat surprisingly and contrary to azide-tagged analogs of similar size, Batt et al. found that, after feeding the commercial MOE reagents Ac_4_GalNAlk **1** and Ac_4_GlcNAlk **2**, the most simple alkyne-tagged UDP-GalNAc and UDP-GlcNAc derivatives, UDP-GalNAlk **3** and UDP-GlcNAlk **4**, are biosynthesized in varying and often low efficiency in mammalian cells (Fig. 1A).^18^ Previous experience by us and Yu et al. on delivering UDP-sugar analogs with an even longer side chains suggested that AGX1-mediated pyrophosphorylation may be a roadblock to the biosynthesis of UDP-GalNAc/GlcNAc derivatives.^16,17^ These longer derivatives could only be delivered through caged sugar-1-phosphates that are of limited stability and tedious to synthesize. We thus sought to investigate if simply enhancing pyrophosphorylation with mut-AGX1 would allow delivery from the readily available reagents Ac_4_GalNAlk **1** and Ac_4_GlcNAlk **2**.

Here, we profile the metabolic fate of the weak MOE reagents Ac_4_GalNAlk **1** and Ac_4_GlcNAlk **2** in order to turn both reagents into highly efficient tools to probe cell surface glycosylation. We find that mut-AGX1 effectively biosynthesizes UDP-GalNAlk **3** and UDP-GlcNAlk **4** with greatly increased efficiency over WT-AGX1 from caged precursors that can thus be used to profile cell surface protein glycosylation. By suppressing GALE-mediated epimerization, we further find that UDP-GalNAlk **3** and UDP-GlcNAlk **4** enter different subsets to azide-tagged analogs, potentially due to differential acceptance by GTs. We show that close monitoring of the biosynthetic fate enables the development of highly effective MOE reagents.

## Results and Discussion

To study the metabolic fate of UDP-GalNAlk **3** and UDP-GlcNAlk **4**, we first assessed *in vitro* whether both reagents are epimerized by GALE (Fig. 1B). Incubation of synthetic **1** with either a wild type (WT), GALE-containing cell lysate or purified GALE led to epimerization to **2**, as detected either by ion-pair HPLC or high performance anion exchange chromatography (HPAEC). A lysate made from GALE-KO cells did not lead to epimerization. We next profiled the suitability of UDP-GalNAlk **3** as a substrate for members of the GalNAc-T family. GalNAc-Ts prime highly abundant mucin-type protein O-GalNAc glycans, and acceptance of **1** would thus correlate with high cell surface labeling efficiency. Synthetic peptides served as acceptor substrates in *in vitro* glycosylation experiments. Compared to the native substrate UDP-GalNAc, UDP-GalNAlk **3** was used with lower but well-measurable efficiency by GalNAc-T1 and T2, and similar efficiency by GalNAc-T7 and T10 (Fig. 1C). The isoenzymes T7 and T10 differ from T1 and T2 in their preference of pre-O-GalNAc-glycosylated substrate peptides, which may hint to the use of UDP-GalNAlk **3** as a GalNAc-T subset-selective substrate.^20^

**Fig. 2:**
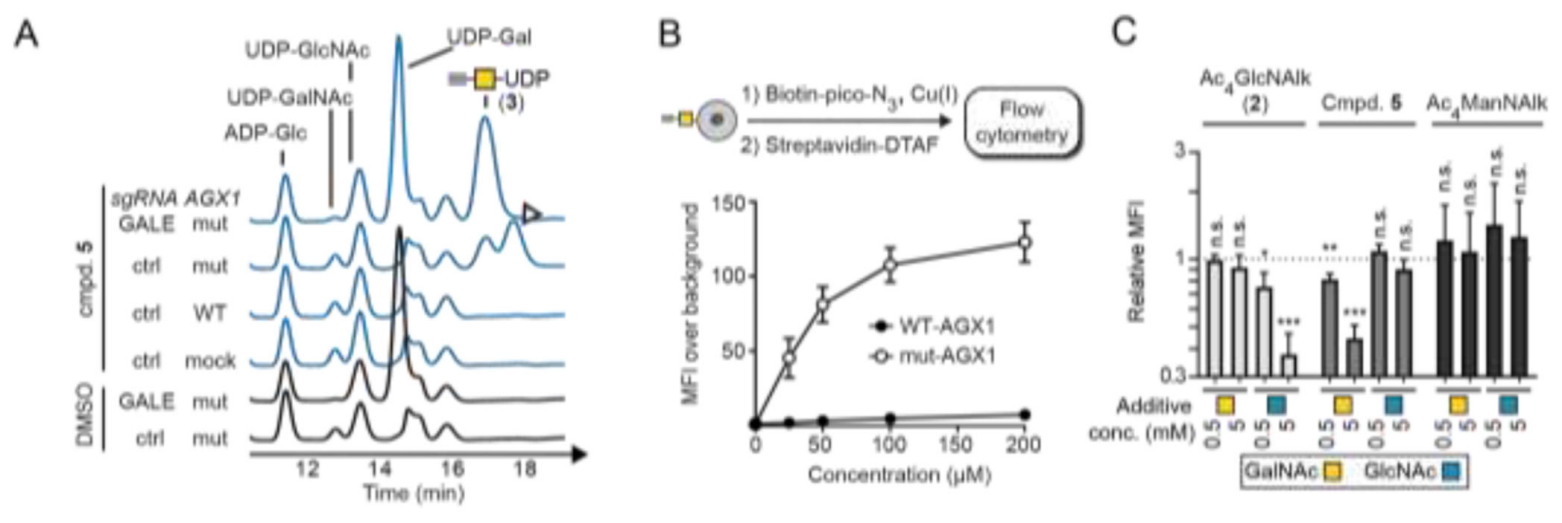
mut-AGX1-mediated biosynthesis of UDP-GalNAlk **3** and cell surface labeling. *A*, metabolite profiling of K-562 cells based on AGX1 expression and presence of GALE by HPAEC. Arrowhead depicts epimerization of UDP-GalNAlk **3** to UDP-GlcNAlk **4**. ADP-glucose was added as an external standard. Data are representative of two independent experiments. *B*, dose response of cell surface labeling of AGX1-stably transfected K-562 cells after feeding **3** as assessed by flow cytometry. Data are means ± SEM as fold increase from DMSO-treated cells from at least three independent experiments. Error bars for WT-AGX1 data are too small to be shown. *C*, competition of cell surface labeling in mut-AGX1-transfected GALE-KO K-562 cells fed with 50 μM caged GalNAlk-1-phosphate **5**, 50 μM Ac_4_GlcNAlk **2** or 10 μM Ac_4_ManNAlk by different concentrations of GalNAc or GlcNAc. Data are means +SD from three independent experiments. Statistical significance was assessed by unpaired, two-tailed t test against labeling experiments without additives (dashed line). Asterisks indicate *P* values: *P < 0.05; **P < 0.01; ***P < 0.001; n.s. non-significant. DTAF = Dichlorotriazinylamino fluorescein; MFI = median fluorescence intensity.

We next studied the biosynthesis of UDP-GalNAlk **3** and UDP-GlcNAlk **4** in cells fed with caged, membrane-permeable precursors. Since AGX1 has been identified as a metabolic bottleneck of other modified GalNAc analogs, we synthesized caged GalNAlk-1-phosphate **5** to specifically probe AGX1-mediated biosynthesis of UDP-GalNAlk **3**.^4,11,17^ We tested UDP-sugar biosynthesis from **3** in K-562 cells with either normal GALE expression or a GALE-knockout and stably transfected with either mut-AGX1, WT-AGX1 or empty vector. HPAEC revealed measurable biosynthesis of both UDP-GalNAlk **3** and UDP-GlcNAlk **4** in the presence of mut-AGX1, but not WT-AGX1 (Fig. 2A, Fig. S2). Levels of UDP-GalNAlk **3** and UDP-GlcNAlk **4** were in the same range as levels or native UDP-GalNAc and UDP-GlcNAc. Free GalNAlk-1-phosphate was detectable in all cases, as observed by comparison with a synthetic standard (Fig. S2). In the absence of GALE, UDP-GlcNAlk was not detectable, indicating that UDP-GalNAlk is biosynthesized by mut-AGX1 and subsequently epimerized by GALE in the cytosol.

We then assessed metabolic cell surface labeling mediated by caged GalNAlk-1-phosphate **5** by flow cytometry. Clickable biotin-picolyl azide was used in non-cytotoxic Cu(I)-click CuAAC conditions followed by streptavidin-DTAF to visualize labeling.^8,21^ The presence of mut-AGX1 led to a dose-dependent increase of fluorescence by up to two orders of magnitude compared to WT-AGX1 (Fig. 2B). Of note, the presence of WT-AGX1 still led to low but discernible cell surface labeling, indicating that UDP-GalNAlk can be biosynthesized at levels that are too low to detect chromatographically. This was especially pronounced in GALE-KO cells in which no endogenous UDP-GalNAc is present to compete with UDP-GalNAlk **3** as substrates of GalNAc-Ts (Fig. 2B, Fig. S3B). A labeling difference of one order of magnitude was observed between cells expressing WT-AGX1 and mut-AGX1 when fed with Ac_4_GlcNAlk **2**, indicating that mut-AGX1 also mediates UDP-GlcNAlk **4** biosynthesis (Fig. S3A). Increasing UDP-GalNAc levels in these cells by supplementing cell culture media with free GalNAc led to a decrease of UDP-GalNAlk **3**-dependent labeling signal (Fig. 2C, Fig. S3C).^17^ Likewise, labeling signal by Ac_4_GlcNAlk **2** was abrogated by addition of free GlcNAc (Fig. 2C). In contrast, labeling by the control compound Ac_4_ManNAlk, an MOE reagent that enters the biosynthetic pathway of the sugar sialic acid, was unchanged irrespective of AGX1 overexpression or addition of free GalNAc or GlcNAc (Fig. 2C, Fig. S3B). Enhancing the levels of native UDP-sugars thus competed out incorporation of GalNAlk and GlcNAlk, but not ManNAlk, into glycoproteins. We concluded that AGX1 is likely a bottleneck in the biosynthesis of both UDP-GalNAlk **3** and UDP-GlcNAlk **4**, impairing metabolic labeling which can be enhanced by stable overexpression of mut-AGX1, but not WT-AGX1. Our data further indicate that Ac_4_GalNAlk **1** exhibits low-level metabolic glycoprotein labeling without mut-AGX1 expression in line with findings of Zaro et al.^7^ UDP-GalNAlk **3** formation is not measurable under these conditions, underlining the highly inefficient biosynthesis of **1** by the GalNAc salvage pathway without mut-AGX1.^18^

As overexpression of mut-AGX1 enabled cell surface labeling from Ac_4_GlcNAlk **2**, GlcNAlk-1-phosphate biosynthesis from the free monosaccharide by NAGK/AGM1 was apparently not a major metabolic bottleneck. We next assessed whether UDP-GalNAlk **3** biosynthesis followed the same principles which would, in turn, allow us to use the readily available MOE reagent Ac_4_GalNAlk **1** instead of caged GalNAlk-1-phosphate **5**. We found that mut-AGX1, but not WT-AGX1, efficiently biosynthesized UDP-GalNAlk **3** and UDP-GlcNAlk **4** from the per-acetylated precursors Ac_4_GalNAlk **1** and Ac_4_GlcNAlk **2**, respectively, in living cells (Fig. S4). We note that the ‘upstream’ precursors Ac_4_GalNAlk **1** and Ac_4_GlcNAlk **2** required longer feeding times (12-16 hours instead of 6-9 hours) than caged GalNAlk-1-phosphate **5** until UDP-sugar biosynthesis could be detected, in line with additional enzymatic reactions being required. At these time points, free GalNAlk-1-phosphate is clearly detectable (Fig. S4) These data indicated that WT-AGX1 is likely the rate-determining step in the biosynthesis of UDP-GalNAlk **3** and UDP-GlcNAlk **4**. Overexpression of mut-AGX1 likely renders the upstream activation steps NAGK/AGM and GALK2 rate-determining.

**Fig. 3:**
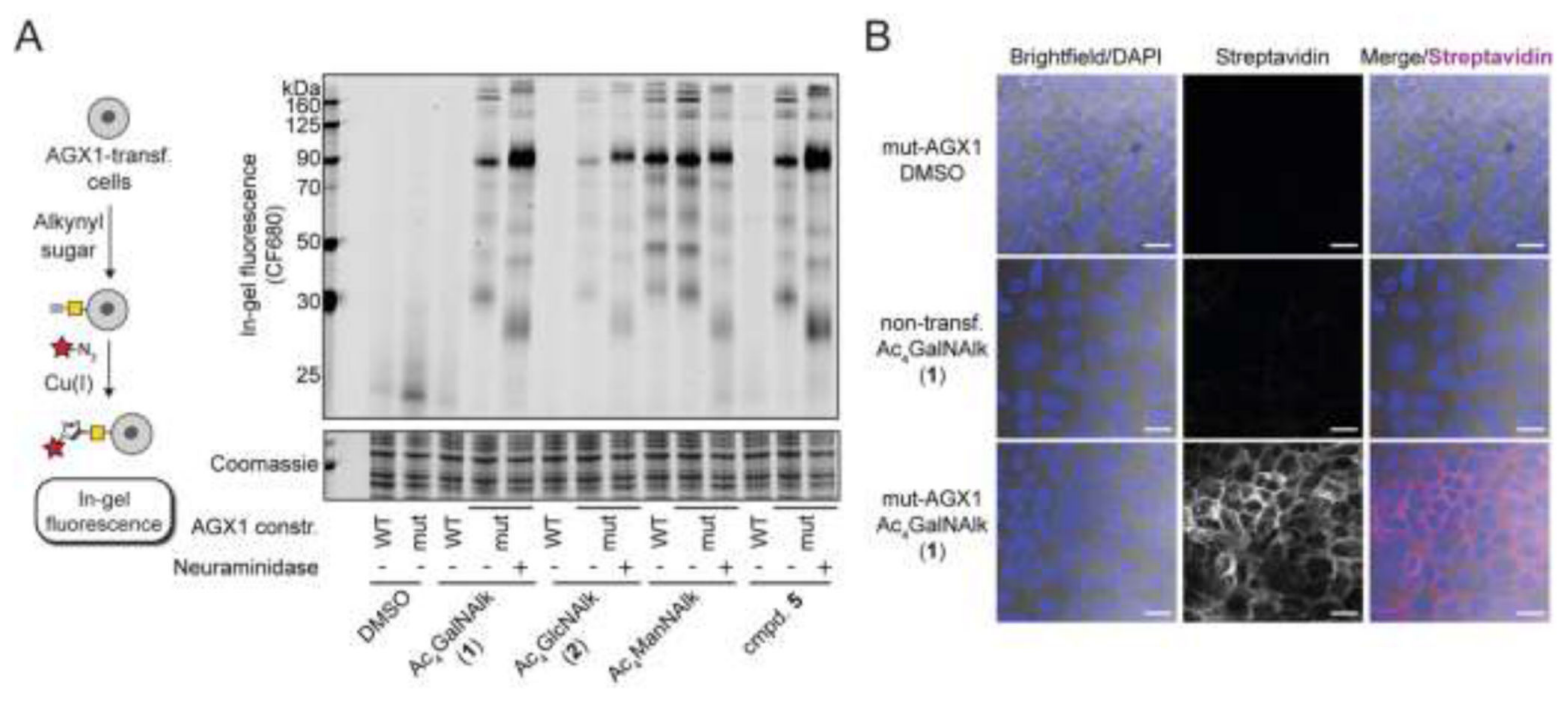
mut-AGX1 enables efficient metabolic labeling with caged precursors of GalNAlk and GlcNAlk. *A*, cell surface labeling of AGX1-transfected K-562 cells fed with 50 μM Ac_4_GalNAlk **1**, 50 μM Ac_4_GlcNAlk **2**, 10 μM Ac_4_ManNAlk or 25 μM caged GalNAlk-1-phosphate **5** as assessed by on-cell CuAAC with the NIR fluorophore CF680-picolyl azide and in-gel fluorescence. cells were treated with the neuraminidase SialEXO before the click reaction where indicated. Data are representative of two independent experiments. *B*, fluorescence microscopy of mut-AGX1-expressing of non-transfected 4T1 cells fed with DMSO or 25 μM Ac_4_GalNAlk **1**, treated with biotin-picolyl-azide under on-cell CuAAC conditions and visualized with Streptavidin-AF647. Data are representative of two independent experiments. Scale bar, 20 μm.

We next visualized the impact of metabolic engineering on glycoprotein labeling by Ac_4_GalNAlk **1** and Ac_4_GlcNAlk **2**. Following the feeding of AGX1-transfected K-562 cells with alkyne-containing monosaccharide precursors, cell surfaces were either treated with a neuraminidase that removes sialic acid from glycoproteins, or left untreated. The living cells were then subjected to CuAAC with the clickable near-infrared fluorophore CF680 picolyl azide, and labeled cell surface glycoproteins were analyzed by in-gel fluorescence.^17^ Under these conditions, the compounds Ac_4_GalNAlk **1**, Ac_4_GlcNAlk **2** and caged GalNAlk-1-phosphate **5** exhibited mut-AGX1-dependent labeling while the control reagent Ac_4_ManNAlk labeled glycoproteins irrespective of the AGX1 construct used (Fig. 3A). Neuraminidase treatment led to an increase of signal in all cases except for Ac_4_ManNAlk-labeled cells, consistent with increased availability of GalNAc- and GlcNAc-carrying alkyne tags towards click reagents when the layer of sialic acid is enzymatically trimmed.^11,17^ While these results suggest that neither GlcNAlk nor GalNAlk enter the sialic acid pool through metabolic interconversion, more detailed experiments are needed to exclude such a metabolic crosstalk.^12^ Of note, the dependence on mut-AGX1 for GalNAlk/GlcNAlk labeling emphasizes that UDP-sugar formation is a prerequisite for efficient labeling, excluding previously reported non-enzymatic cysteine glycosylation that typically happens under very high concentrations of per-acetylated sugars.^22,23^ We next visualized glycocalyx labeling by fluorescence microscopy. We chose adherent murine 4T1 cells to allow for straightforward imaging and further demonstrate the robustness of our approach. Clickable biotin picolyl azide and Streptavidin-AF647 readily detected a large enhancement of Ac_4_GalNAlk **1**-mediated cell surface labeling in 4T1 cells stably expressing mut-AGX1 over non-transfected cells (Fig. 3B). In contrast, cell surface labeling by the AGX1-independent MOE reagent Ac_4_ManNAlk was remained unchanged upon mut-AGX1 transfection (Fig. S5).

**Fig. 4:**
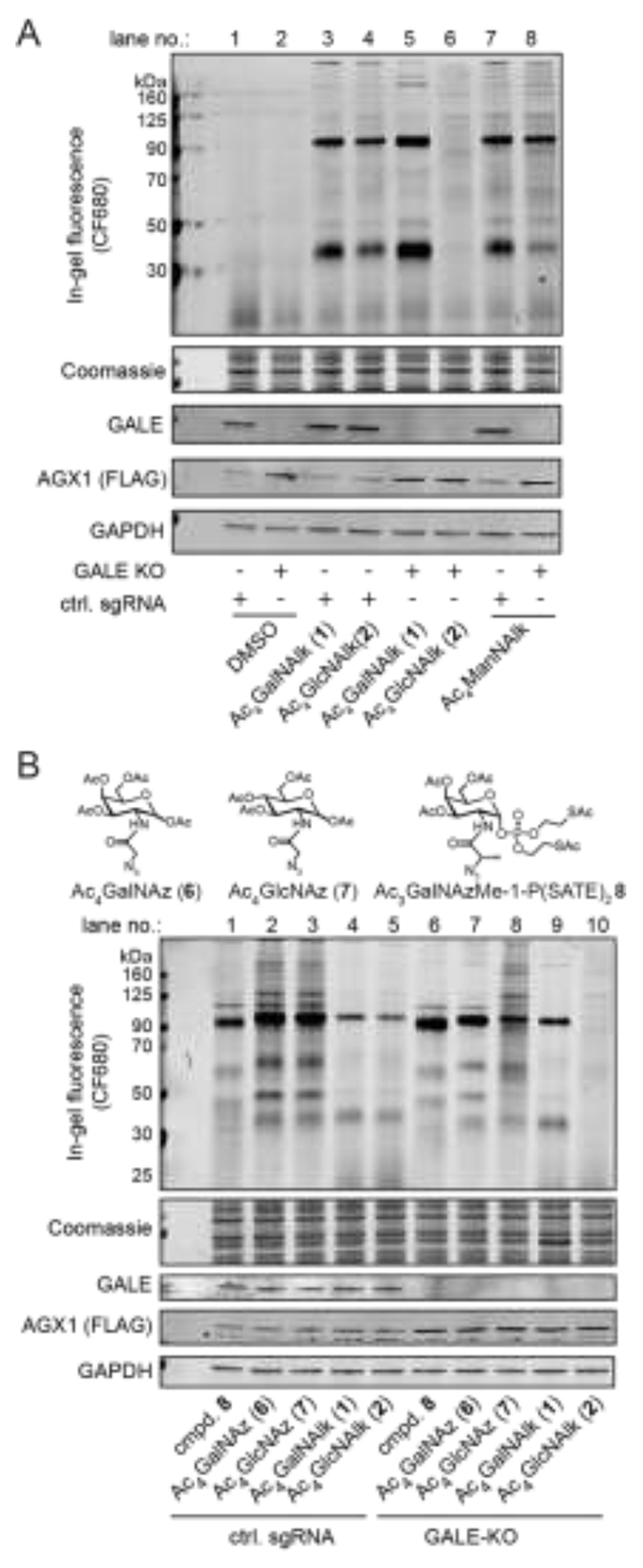
GalNAlk and GlcNAlk-mediated labeling of glycoprotein subsets. *A*, cell surface labeling of mut-AGX1-transfected K-562 GALE-KO or control sgRNA-expressing cells fed with 10 μM Ac_4_GalNAlk **1**, 50 μM Ac_4_GlcNAlk **2**, or 10 μM Ac_4_ManNAlk as assessed by on-cell click chemistry and in-gel fluorescence. Data are representative of two independent experiments. *B*, comparison of cell surface labeling of mut-AGX1-transfected K-562 GALE-KO or control sgRNA-expressing cells fed with 10 μM Ac_4_GalNAlk **1**, 50 μM Ac_4_GlcNAlk **2**, 3 μM Ac_4_GalNAz **6**, 8 μM Ac_4_GlcNAz **7** or 100 μM caged GalNAzMe-1-phosphate **8**. Data are representative of two independent experiments.

Due to GALE-mediated interconversion of UDP-GalNAlk and UDP-GlcNAlk, the glycoprotein profile labeled by both MOE reagents Ac_4_GalNAlk **1** and Ac_4_GlcNAlk **2** is identical (Fig. 3A). To assess the contribution of each UDP-sugar to signal, we profiled the glycoprotein patters in GALE-KO cells that functionally separate UDP-GalNAlk **3** and UDP-GlcNAlk **4** (Fig 4A). Cells were grown in GalNAc- containing media to maintain native levels of metabolites such as UDP-GalNAc, allowing for comparison with GALE-expressing control cells when all cell lines were transfected with mut-AGX1. DMSO feeding did not lead to discernible labeling (Fig. 4A, lanes 1 and 2).

While GALE-expressing control cells displayed identical labeling patterns when fed with either Ac_4_GalNAlk **1** or Ac_4_GlcNAlk **2** (Fig. 4A, lanes 3 and 4), GALE-KO had a striking effect on labeling patterns. In GALE-KO cells, Ac_4_GalNAlk **1** contributed highly intense glycoprotein bands at approx. 100 kDa and 40 kDa (Fig. 4A, lane 5), while Ac_4_GlcNAlk **2** contributed a diffuse pattern of lower overall intensity (Fig. 4A, lane 6). These results suggested that separating the UDP-GalNAlk **3** and UDP- GlcNAlk **4** pools led to labeling of different subsets of glycoproteins. In contrast, feeding Ac_4_ManNAlk led to similar band patterns in both GALE-expressing and GALE-KO cell lines (Fig. 4A, lanes 7 and 8), indicating that sialylation is not affected by GALE-KO. We speculated that the intense bands labeled by UDP-GalNAlk **3** (Fig. 4A, lanes 3, 4 and 5) are highly GalNAc-glycosylated mucin domain-containing glycoproteins. To test this notion, we subjected GalNAlk-fed and CF680-picolyl azide labeled K-562 cells to different concentrations of the mucin protease StcE or the more promiscuous O-glycoprotease OpeRATOR (Fig. S6).^24^ Treatment with both proteases led to a decrease of cell surface glycoprotein signal in a dose-dependent manner, while signal was recovered as fluorescently labeled broad bands of lower molecular weight in the supernatant. Several glycoprotein bands were digested by OpeRATOR, but not StcE, indicating that labeling of non-mucins containing O-GalNAc glycans was observed. These data confirm that GalNAlk enters mucin domain-containing proteins and other O-GalNAc-glycosylated proteins.

We next compared the Ac_4_GalNAlk **1** and Ac_4_GlcNAlk **2** labeling band patterns in GALE-KO or control cells with previously characterized, azide-containing MOE reagents Ac_4_GalNAz **6** and Ac_4_GlcNAz **7** (Fig. 4B). Both reagents are converted to azide-tagged UDP-GlcNAc/GalNAc analogs that are interconvertible by GALE.^10–12^ We further used the O-GalNAc-specific reagent Ac_3_GalNAzMe-1-P(SATE)_2_ **8**, a precursor to an epimerization-resistant, azide-tagged UDP-GalNAc analog that is not a substrate for GALE in the living cell.^11^ To ensure that band patterns are comparable between azide- and alkyne-tagged monosaccharides, we used the same NIR-fluorophore CF680 with either alkyne or picolyl azide groups for CuAAC. Compound **8** showed the band pattern attributable to O-GalNAc glycosylation in GALE-containing and GALE-KO cells (Fig. 4B, lanes 1 and 6). Ac_4_GalNAz **6**/Ac_4_GlcNAz **7** (Fig. 4B, lanes 2 and 3) labeled the same band pattern in GALE-containing cells, consistent with interconversion of azide-tagged UDP-sugar pools.^10,11^ This labeling pattern was somewhat different from the pattern observed after feeding GALE-containing cells Ac_4_GalNAlk **1/**Ac_4_GlcNAlk **2** (Fig. 4B, lanes 4 and 5), with more bands being visible with azide-tagged monosaccharide analogs. These findings can be explained by UDP-GalNAz being a better substrate for the commonly expressed glycosyltransferases GalNAc-T1 and T2 than UDP-GalNAlk **3** (Fig. 1C).^11,19^ Upon GALE-KO, Ac_4_GalNAz **6** and Ac_4_GlcNAz **7** led to different band patterns as reported (Fig. 4B, lanes 7 and 8).^11^ In comparison, The Ac_4_GalNAlk **1**-labeled band pattern in GALE-KO cells resembled only a subset of the pattern seen from Ac_4_GalNAz **6** or compound **8** feeding (Fig. 4B, lane 9), indicating that UDP-GalNAlk **3** labels a subset of O-GalNAc glycoproteins. Finally, Ac_4_GlcNAlk **2** exhibited diffuse labeling pattern in GALE-KO cells (Fig. 4B, lane 10) similar to the azide-tagged counterpart Ac_4_GalNAz **7**. Taken together, these data suggest that UDP-GalNAlk **3** and UDP-GlcNAlk **4** label different sets of glycoproteins but are interconnected by GALE in the living cell. Structurally simple azide- and alkyne-tagged GalNAc/GlcNAc derivatives label particular glycoprotein subsets and should thus serve as orthogonal, but potentially complementary MOE reagents in the presence of mut-AGX1. Western Blot analysis with an antibody against AGX1 indicated that our expression constructs lead to an approx. 2-3-fold overexpression, suggesting that metabolic engineering does not require abundant expression levels of mut-AGX1.

We have shown that comprehensive metabolic profiling can turn weak MOE reagents into efficient chemical biology tools to profile cellular glycosylation. The expression of mut-AGX1 enhances labeling by Ac_4_GalNAlk **1** and Ac_4_GlcNAlk **2** by orders of magnitude, substantially expanding the toolbox for glycobiology. While our approach relies cell transfection, the plasmids we use are based on transposase- mediated stable integration which is compatible even with hard-to-transfect cell lines and more complex model systems such as organoids.^11^ Our work focused on improving metabolic labeling efficiency with Ac_4_GalNAlk **1**/Ac_4_GlcNAlk **2**. While we showed that the corresponding activated sugars UDP-GalNAlk **3**/UDP-GlcNAlk **4** can be incorporated in GalNAc- and GlcNAc-containing glycoconjugates, we did not assess their fine specificity for certain subtypes of glycans. We and others have previously focused on assessing such specificity for similar MOE reagents,^11,25^ and further studies extending this work are underway.

## Supplementary Material

Fig. S1

## Figures and Tables

**Figure:**
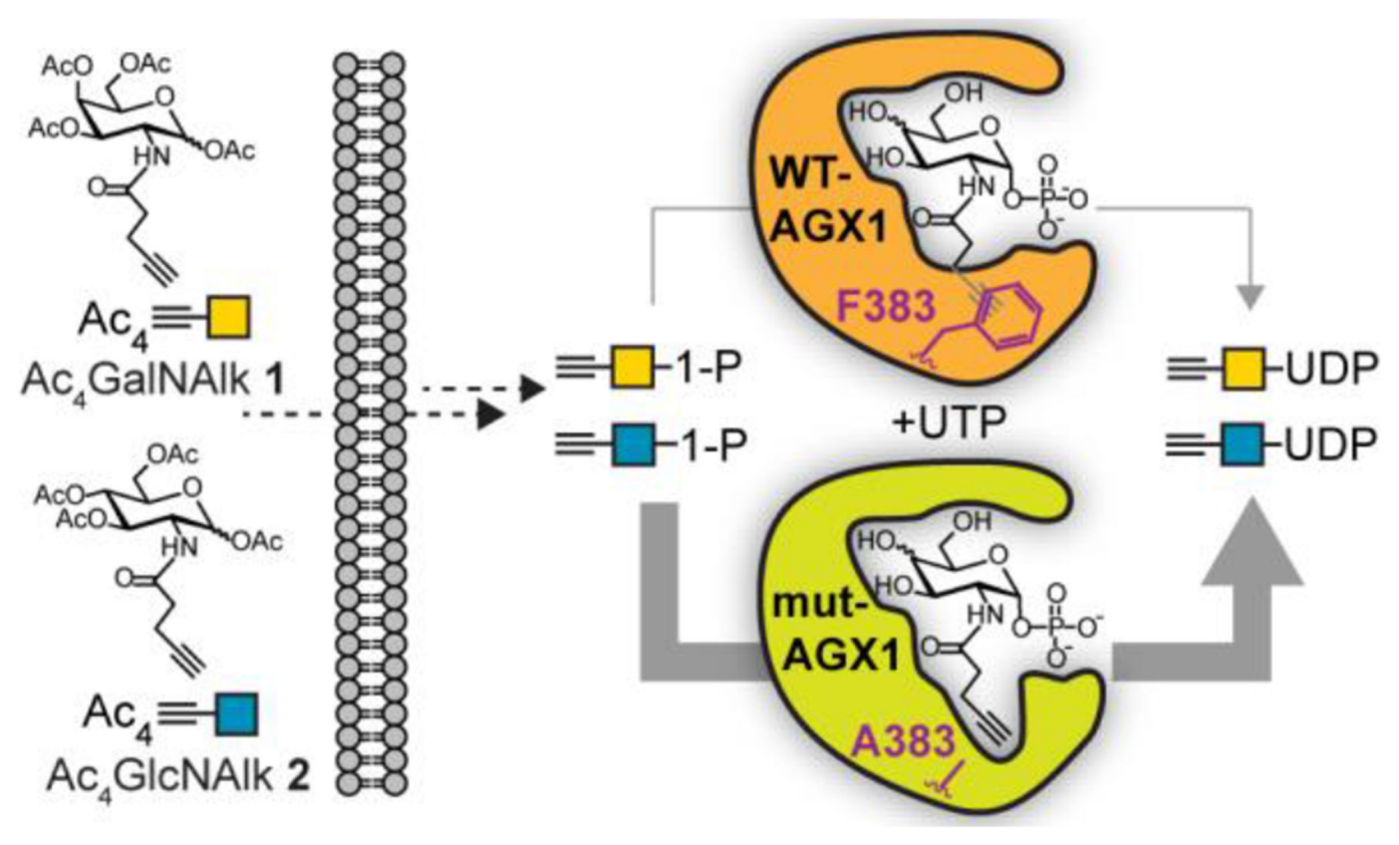
For Table of Contents only

## References

[1] Sletten EM, Bertozzi CR (2009). Bioorthogonal Chemistry: Fishing for Selectivity in a Sea of Functionality. Angew Chem Int Ed.

[2] Parker CG, Pratt MR (2020). Click Chemistry in Proteomic Investigations. Cell.

[3] Zol-Hanlon MI, Schumann B (2020). Open Questions in Chemical Glycobiology. Commun Chem.

[4] Cioce A, Malaker SA, Schumann B (2021). Generating Orthogonal Glycosyltransferase and Nucleotide Sugar Pairs as Next-Generation Glycobiology Tools. Curr Opin Chem Biol.

[5] Mahal LK, Yarema KJ, Bertozzi CR (1997). Engineering Chemical Reactivity on Cell Surfaces through Oligosaccharide Biosynthesis. Science.

[6] Hang HC, Yu C, Pratt MR, Bertozzi CR (2004). Probing Glycosyltransferase Activities with the Staudinger Ligation. J Am Chem Soc.

[7] Zaro BW, Yang YY, Hang HC, Pratt MR (2011). Chemical Reporters for Fluorescent Detection and Identification of O-GlcNAc-Modified Proteins Reveal Glycosylation of the Ubiquitin Ligase NEDD4-1. Proc Natl Acad Sci U S A.

[8] Besanceney-Webler C, Jiang H, Zheng T, Feng L, Soriano Del Amo D, Wang W, Klivansky LM, Marlow FL, Liu Y, Wu P (2011). Increasing the Efficacy of Bioorthogonal Click Reactions for Bioconjugation: A Comparative Study. Angew Chem Int Ed.

[9] Peneff C, Ferrari P, Charrier V, Taburet Y, Monnier C, Zamboni V, Winter J, Harnois M, Fassy F, Bourne Y (2001). Crystal Structures of Two Human Pyrophosphorylase Isoforms in Complexes with UDPGlc(Gal)NAc: Role of the Alternatively Spliced Insert in the Enzyme Oligomeric Assembly and Active Site Architecture. EMBO J.

[10] Boyce M, Carrico IS, Ganguli AS, Yu SH, Hangauer MJ, Hubbard SC, Kohler JJ, Bertozzi CR (2011). Metabolic Cross-Talk Allows Labeling of O-Linked β-N-Acetylglucosamine-Modified Proteins via the N-Acetylgalactosamine Salvage Pathway. Proc Natl Acad Sci U S A.

[11] Debets MF, Tastan OY, Wisnovsky SP, Malaker SA, Angelis N, Moeckl LKR, Choi J, Flynn H, Wagner LJS, Bineva-Todd G, Antonopoulos A (2020). Metabolic Precision Labeling Enables Selective Probing of O-Linked N-Acetylgalactosamine Glycosylation. Proc Natl Acad Sci U S A.

[12] Shajahan A, Supekar NT, Wu H, Wands AM, Bhat G, Kalimurthy A, Matsubara M, Ranzinger R, Kohler JJ, Azadi P (2020). Mass Spectrometric Method for the Unambiguous Profiling of Cellular Dynamic Glycosylation. ACS Chem Biol.

[13] Hang HC, Yu C, Kato DL, Bertozzi CR (2003). A Metabolic Labeling Approach toward Proteomic Analysis of Mucin-Type O-Linked Glycosylation. Proc Natl Acad Sci U S A.

[14] Woo CM, Iavarone AT, Spiciarich DR, Palaniappan KK, Bertozzi CR (2015). Isotope-Targeted Glycoproteomics (IsoTaG): A Mass-Independent Platform for Intact N- and O-Glycopeptide Discovery and Analysis. Nat Methods.

[15] Pouilly S, Bourgeaux V, Piller F, Piller V (2012). Evaluation of Analogues of GalNAc as Substrates for Enzymes of the Mammalian GalNAc Salvage Pathway. ACS Chem Biol.

[16] Yu SH, Boyce M, Wands AM, Bond MR, Bertozzi CR, Kohler JJ (2012). Metabolic Labeling Enables Selective Photocrosslinking of O-GlcNAc-Modified Proteins to Their Binding Partners. Proc Natl Acad Sci U S A.

[17] Schumann B, Malaker SA, Wisnovsky SP, Debets MF, Agbay AJ, Fernandez D, Wagner LJS, Lin L, Li Z, Choi J, Fox DM (2020). Bump-and-Hole Engineering Identifies Specific Substrates of Glycosyltransferases in Living Cells. Mol Cell.

[18] Batt AR, Zaro BW, Navarro MX, Pratt MR (2017). Metabolic Chemical Reporters of Glycans Exhibit Cell-Type-Selective Metabolism and Glycoprotein Labeling. ChemBioChem.

[19] Choi J, Wagner LJS, Timmermans SBPE, Malaker SA, Schumann B, Gray MA, Debets MF, Takashima M, Gehring J, Bertozzi CR (2019). Engineering Orthogonal Polypeptide GalNAc-Transferase and UDP-Sugar Pairs. J Am Chem Soc.

[20] de las Rivas M, Lira-Navarrete E, Gerken TA, Hurtado-Guerrero R (2019). Polypeptide GalNAc-Ts: From Redundancy to Specificity. Curr Opin Struct Biol.

[21] Uttamapinant C, Tangpeerachaikul A, Grecian S, Clarke S, Singh U, Slade P, Gee KR, Ting AY (2012). Fast, Cell-Compatible Click Chemistry with Copper-Chelating Azides for Biomolecular Labeling. Angew Chem Int Ed.

[22] Qin W, Qin K, Fan X, Peng L, Hong W, Zhu Y, Lv P, Du Y, Huang R, Han M, Cheng B (2018). Artificial Cysteine S-Glycosylation Induced by Per-O-Acetylated Unnatural Monosaccharides during Metabolic Glycan Labeling. Angew Chem Int Ed.

[23] Qin K, Zhang H, Zhao Z, Chen X (2020). Protein S-Glyco-Modification through an Elimination-Addition Mechanism. J Am Chem Soc.

[24] Malaker SA, Pedram K, Ferracane MJ, Bensing BA, Krishnan V, Pett C, Yu J, Woods EC, Kramer JR, Westerlind U, Dorigo O (2019). The Mucin-Selective Protease StcE Enables Molecular and Functional Analysis of Human Cancer-Associated Mucins. Proc Natl Acad Sci U S A.

[25] Pedowitz NJ, Pratt MR (2021). Design and synthesis of metabolic chemical reporters for the visualization and identification of glycoproteins. RSC Chem Biol.

