# Supplementary material for: Optimization of metabolic oligosaccharide engineering with Ac4GalNAlk and Ac4GlcNAlk by an engineered pyrophosphorylase
Source: ACS Chem Biol. Author manuscript; Available in PMC 2021 Oct 19. (PMC8501146; doi:10.1021/acschembio.1c00034)
Supplement: Fig. S1 [file EMS122676-supplement-Fig__S1.pdf]

Optimization of metabolic oligosaccharide engineering with Ac_4GalNAlk and Ac_4GlcNAlk by an engineered pyrophosphorylase

Supporting Information

Supporting Figures
Experimentals
Characterization of synthetic compounds

Anna Cioce[a,b,¶], Ganka Bineva-Todd[b,¶], Anthony J. Agbay[c], Junwon Choi[c,£], Thomas M. Wood[c,$], Marjoke F. Debets[c,#], William M. Browne[a,b], Holly L. Douglas[d], Chloe Roustan[e], Svend Kjaer[e], Jacob T. Bush[f], Carolyn R. Bertozzi[c,g], Benjamin Schumann[a,b,*]

[a]Department of Chemistry, Imperial College London, 80 Wood Lane, W12 0BZ, London, United Kingdom.
[b]The Chemical Glycobiology Laboratory, The Francis Crick Institute, 1 Midland Rd, NW1 1AT London, United Kingdom.
[c]Department of Chemistry, Stanford University, Stanford, CA 94305, USA.
[d]Mycobacterial Metabolism and Antibiotic Research Laboratory, The Francis Crick Institute, 1 Midland Rd, NW1 1AT London, United Kingdom.
[e]Structural Biology Science Technology Platform, The Francis Crick Institute, NW1 1AT London, United Kingdom.
[f]GlaxoSmithKline, Gunnels Wood Road, Stevenage, Hertfordshire, SG1 2NY UK.
[g]Howard Hughes Medical Institute, 380 Roth Way, Stanford, CA 94305, USA.

[£]Current address: Korea Institute of Science and Technology, Hwarangro 14-gil 5, Seongbuk-gu, Seoul, 02792, Republic of Korea.

[$]Current address: Biological Chemistry Group, Institute of Biology Leiden, Leiden University, Sylvius Laboratories, Sylviusweg 72, 2333 BE Leiden, The Netherlands.

[#]Current address: Lilly Research Laboratories, Eli Lilly and Company, Indianapolis, IN 46285, USA.

[¶]These authors contributed equally.

*Correspondence should be addressed to: b.schumann@imperial.ac.uk.

Supporting Figures

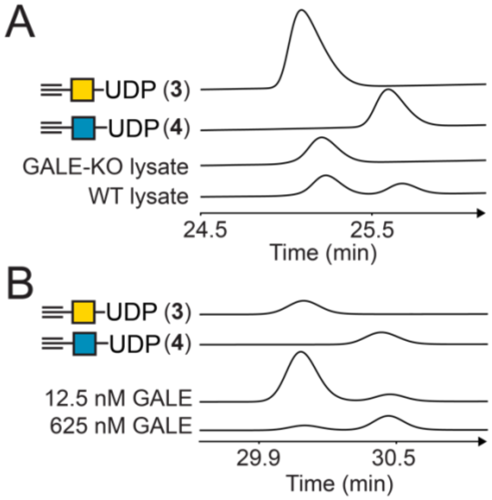

Fig. S1: *in vitro* epimerization of UDP-GalNAlk **3** to UDP-GlcNAlk **4**. *A*, reaction using a GALE-containing cell lysate or a GALE-KO lysate as a control as assessed by HPAEC. Traces are the same as Fig. 1B but include synthetic standards. *B*, reaction using two concentrations of purified GALE as assessed by ion-pair HPLC.[1]

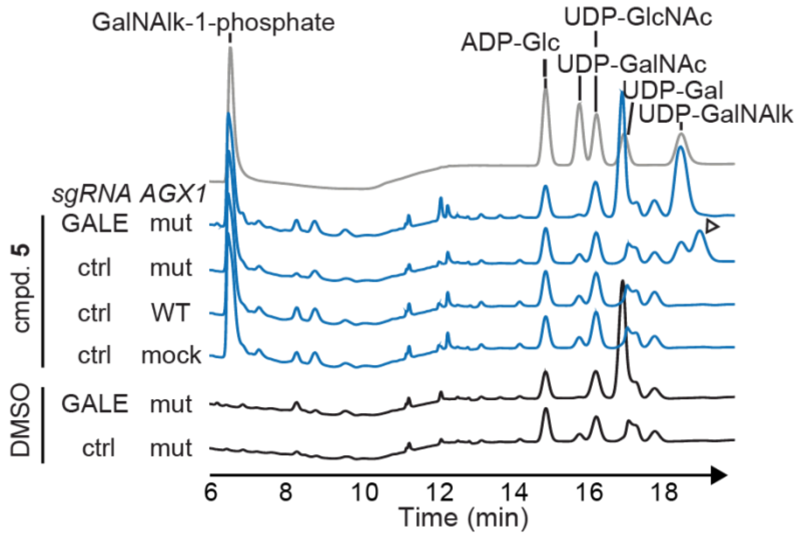

Fig. S2: mut-AGX1-mediated biosynthesis of UDP-GalNAlk **3** and cell surface labeling. Metabolite profiling of K-562 cells based on AGX1 expression and presence of GALE by HPAEC. Grey trace represents standards. Arrowhead depicts epimerization of UDP-GalNAlk **3** to UDP-GlcNAlk **4**. Data are representative of two independent experiments.

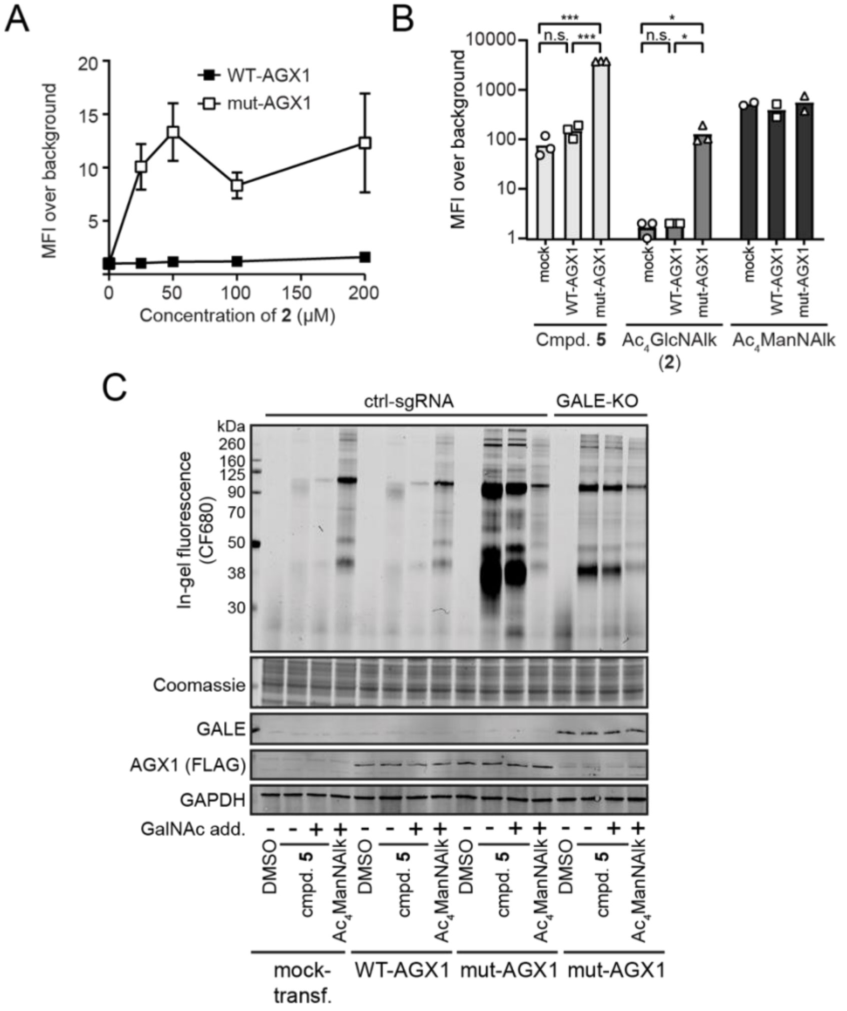

Fig. S3: mut-AGX1-mediated biosynthesis of UDP-GalNAlk **3** and UDP-GlcNAlk **4**, and cell surface labeling. *A*, dose response of cell surface labeling of AGX1-stably transfected K-562 cells after feeding Ac_4GlcNAlk **2** and flow cytometry. Data are means ± SEM as fold increase from DMSO-treated cells from at least three biological replicates. Error bars for WT-AGX1 data are too small to be shown. *B*, mut-AGX1-mediated cell surface labeling in GALE-KO K-562 cells fed with 50 µM caged GalNAlk-1-phosphate **5**, 50 µM Ac_4GlcNAlk **2** or 10 µM Ac_4ManNAlk as fold increase over DMSO-fed cells, as assessed by flow cytometry. Data are individual values and means from three or two independent experiments. Statistical significance was assessed by One-way ANOVA with Tukey's post-hoc test. Asterisks indicate *P* values: **P* < 0.05; ****P* < 0.001; n.s. non-significant. *C*, cell surface labeling of AGX1-transfected K-562 GALE-KO or control sgRNA-transfected cells fed with 20 µM caged GalNAlk-1-phosphate **5** or 10 µM Ac_4ManNAlk as assessed by on-cell CuAAC and in-gel fluorescence as in Fig. 3. Cell media were supplemented with 0.5 mM GalNAc as indicated. Data are representative of two independent experiments.

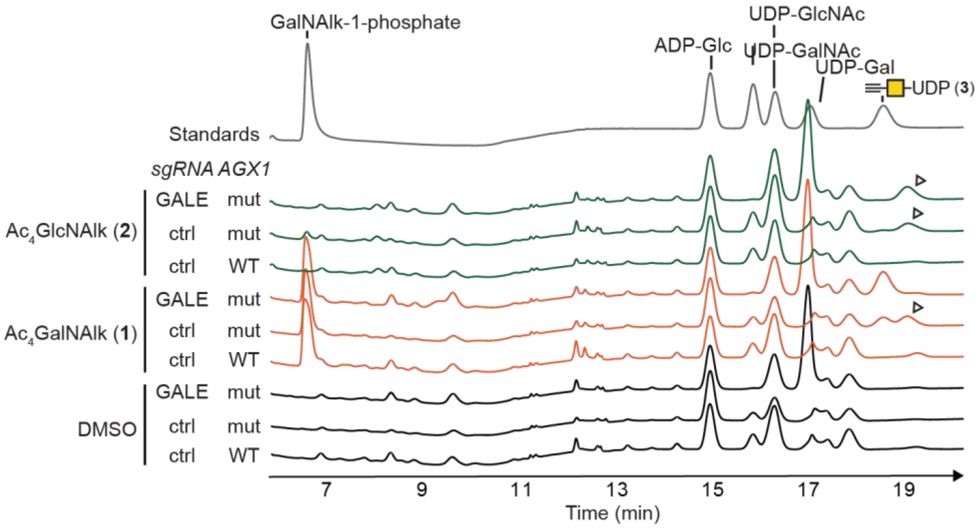

Fig. S4: metabolite profiling of K-562 cells based on AGX1 expression and presence of GALE by HPAEC after feeding Ac_4GalNAlk **1** or Ac_4GlcNAlk **2**. Grey trace represents standards. Arrowheads depict UDP-GlcNAlk **4**. Data are representative of two independent experiments.

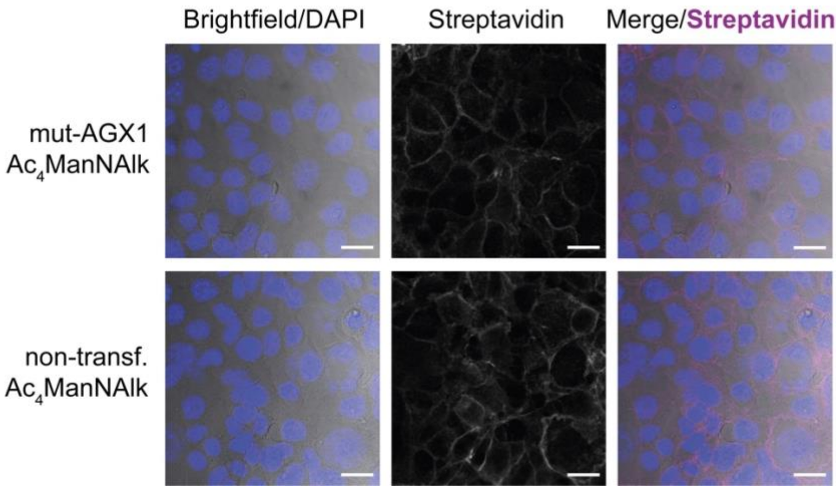

Fig. S5: Fluorescence microscopy of mut-AGX1- or non-transfected 4T1 cells fed with 10 µM $Ac_4ManNAlk$, treated with biotin-picolyl azide under on-cell CuAAC conditions and visualized with Streptavidin-AF647. Scale bar, 20 µm.

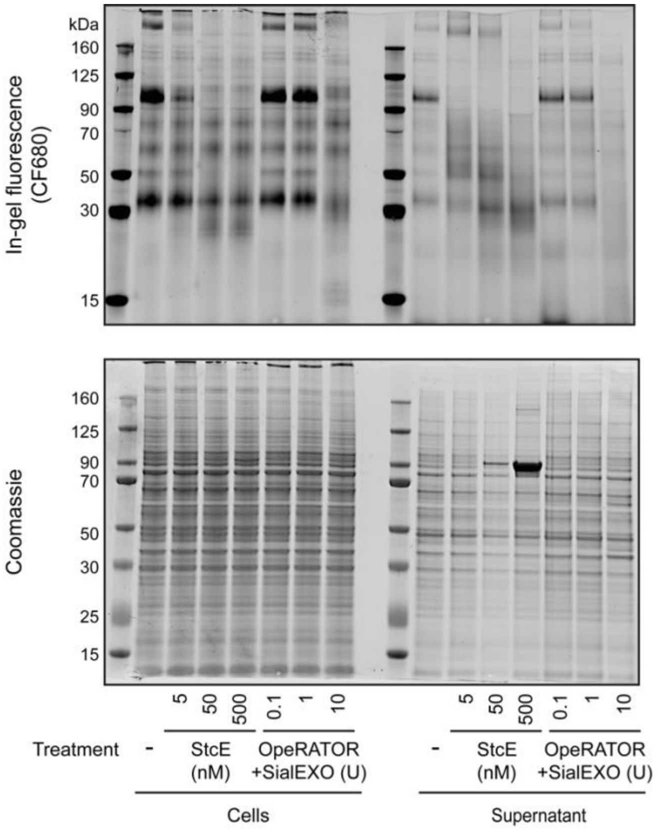

Fig. S6: Glycoprotease-mediated abrogation of alkyne-labeled cell surface glycoproteins. Mut-AGX1-transfected K-562 cells were fed with caged GalNAlk-1-phosphate **5**, subjected to on-cell CuAAC with CF680-picolyl azide as in Fig. 3A, and treated with different concentrations of StcE or OpeRATOR/SialEXO. Cell lysates and supernatant were analyzed by in-gel fluorescence. Data are from one experiment.

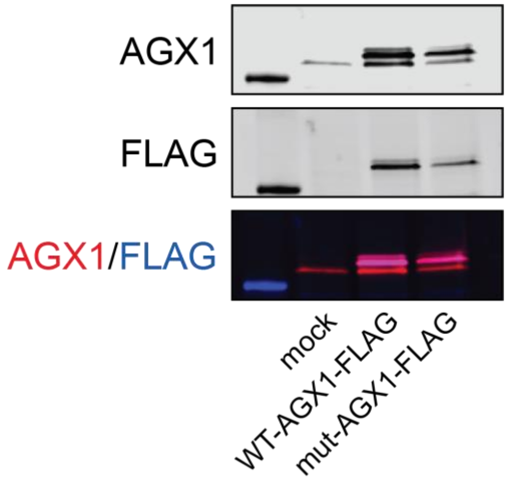

Fig. S7: Expression analysis of AGX1. Cells transfected with empty plasmid (mock), pSBbi-AGX1^{WT} (WT-AGX1-FLAG) or pSBbi-AGX1^{F383A} (mut-AGX1-FLAG) were lysed and AGX1 expression analyzed by Western Blot using antibodies against AGX1 or the FLAG tag. AGX1-FLAG constructs probably appear as double bands due to the presence of a 2A peptide.

Experimentals

In vitro epimerization

Epimerization of UDP-GalNAlk was assessed as previously established.[1] Briefly, reactions were run in 25 µL volume, containing either cell lysates (12 µg protein) from GALE-KO K-562 cells or control cells expressing GALE, or purified human GALE (12.5 nM or 625 nM) in 25 mM Glycine-HCl (pH 8.7), 200 µM NAD and 250 µM UDP-GalNAlk and 5 mM sodium pyruvate (only for reactions with cell lysates). Reactions were run for 30 min or overnight with purified GALE or cell lysates, respectively, at 37 °C. After that, reactions were diluted with 75 µL water and cooled to 4 °C. Reactions with purified GALE were run on a 1260 HPLC with diode array detector using a Poroshell 120, EC-C18, 2.7 µm, 3.0 x 150 mm column (Agilent, Santa Clara, USA). Reactions performed with cell lysates were run on an ICS-6000 with a quaternary pump, a conductivity detector, an ERD 500 ion suppressor and an EGC 500 KOH eluent generator cartridge using an AS11-HC-4µm 250 mm column with an AG11-HC-4µm guard column (Thermo Fisher, Hemel Hempstead, UK). Solvents for HPLC were A = 100 mM potassium phosphate, pH 6.4, 8 mM tetrabutylammonium bisulfate; B = 80% A, 20% acetonitrile. Gradient was 0 min 0% B; 19 min 25% B; 32 min 100% B; 34 min 100% B; 35 min 0% B; 46 min 0% B. Eluent for ion chromatography (in mM KOH) was 0 min 1 mM; 3 min 1 mM; 8 min 31 mM; 13 min 36 mM; 38 min 70 mM; 43 min 100 mM; 48 min 100 mM; 53 min 1 mM. Commercial or synthetic standards (200-500 µM) were used as controls for chromatography.

Peptide glycosylation

HPLC was performed on a 1100 series HPLC system (Agilent). LC-MS experiments were performed on a 1260 Infinity HPLC attached to a 6120 Quadrupole mass spectrometer (Agilent). Poroshell 120 EC-C18, 2.7 µm, 4.6 × 50 mm analytical LC columns (Agilent) were used for HPLC and LC-MS.

In vitro glycosylation was performed as previously reported.[2,3] Soluble GalNAc-T1, T2, T7 and T10 enzymes were expressed and used previously.[2,3] For T2, T7 and T10, chromophore-tagged, isoenzyme-optimized Peptide-1 [(DAA)HN-GAGAPGPTPGPAGAGK; DAA = 2,4-dinitrophenyl-5-L-alanine amide] or Peptide-2 [(DAA)HN-GTT(α-D-GalNAc)PSPVPTTSTTSAP] were used as substrates with an HPLC-based assay to assess conversion.[2,3] For T1, EA2 peptide (Anaspec, Fremont, USA) was used as substrate with LCMS-based detection of conversion. All reaction mixtures contained 20.8 mM Tris-HCl (pH 7.4), 50 mM NaCl, 10 mM $MnCl_2$, 12.5% glycerol, GalNAc-T enzymes (80 nM T1, 25 nM T2, 50 nM T7, 60 nM T10), 250 µM UDP-GalNAlk and 50 µM peptide substrate in 50 µL volume. Reactions were carried out for 2 h (T1) or 1 h (T2, T7, T10), quenched with150 mM EDTA pH 8.0 (25 µL) and analyzed by HPLC or LCMS. The gradients used as percentage of acetonitrile in water with 0.1% (v/v) formic acid were for Peptide-1/UDP-GalNAc 0 min 21.5%; 35 min 21.5%; 36 min 100%; 40 min 100%; 41 min 21.5%; 45 min 21.5%; for Peptide-1/UDP-GalNAlk 0 min 21%; 30 min 21%; 31 min 100%; 35 min 100%; 36 min 21%; 40 min 21%; for Peptide-2/UDP-GalNAc or Peptide-2/UDP-GalNAlk 0 min 22.5%; 25 min 22.5%; 26 min 100%; 30 min 100%; 31 min 22.5%; 35 min 22.5%. Control glycosylation data using UDP-GalNAc were reported previously.[1]

For reactions containing GalNAc-T1, extracted ion chromatograms (EIC) were generated of singly and doubly glycosylated species, integrated and related to the intensity of unglycosylated peptide. The m/z values $[M+2H]^{2+}$ used were 658.71 (EA2), 760.31 (EA2-GalNAc), 942.99 (EA2-2xGalNAlk). Of note, total intensities of peptide and glycopeptide species inversely correlated with the abundance of doubly

glycosylated species. As doubly glycosylated species were mainly present in glycosylations with UDP-GalNAc, the abundance of unglycosylated peptides was likely overestimated for glycosylations using UDP-GalNAlk.

Plasmids and Cell lines

The plasmids pSBtet-AGX1^{WT} or pSBtet-AGX1^{F383A} were prepared previously.[3] AGX1^{WT} and AGX1^{F383A} were introduced into the plasmid pSBbi using a previously reported cloning strategy.[1] The pSBbi plasmid was a gift from Eric Kowarz (Addgene plasmid #60514; http://n2t.net/addgene:60514; RRID:Addgene_60514) (Addgene, Watertown, USA).[4]

All cells were screened for contamination by mycoplasma and other cell lines by the Crick Cell Services Science Technology Platform. K-562 cells were propagated in RPMI (Thermo Fisher) with 10% (v/v) FBS, penicillin (100 U/mL) and streptomycin (100 µg/mL). K-562 cells stably transfected with pSBtet-AGX1^{WT} or pSBtet-AGX1^{F383A} have been prepared previously.[3] K-562 cells were stably transfected with pSBbi-AGX1 constructs or empty pSBbi-GH using Lipofectamine LTX (Thermo) according to the manufacturer's instructions, with a 20:1 (m/m) mixture of pSBbi and pCMV(CAT)T7-SB100 plasmid DNA. After 24 h, cells were harvested and selected in growth medium containing 150 µg/mL hygromycin B (Thermo) for 7-10 days to obtain stable cells. 4T1(GFP) cells were a gift from Ilaria Malanchi (Francis Crick Institute) and maintained in DMEM (Thermo Fisher) with 10% (v/v) FBS, penicillin (100 U/mL) and streptomycin (100 µg/mL. Cells were transfected with pSBbi-AGX1^{F383A} plasmid using Lipofectamine 3000 (Thermo Fisher) according to the manufacturer's specifications, with a 20:1 (m/m) mixture of pSBbi-AGX1^{F383A} and pSB100 plasmid DNA. After 24 h, cells were harvested and selected in growth medium containing 100 µg/mL hygromycin B (Thermo) for 7-10 days to obtain stable cells.

Analysis of nucleotide-sugar biosynthesis by High Performance Anion Exchange Chromatography

Cells expressing FLAG-tagged AGX1 constructs (5 million K-562 or K-562 GALE-KO cells stably transfected with pSBtet-AGX1^{WT} or pSBtet-AGX1^{F383A} in 4 mL growth medium without hygromycin B) were fed with 100 µM (from a 100 mM stock solution in DMSO) caged GalNAlk-1-phosphate analog **5**, Ac_4GalNAlk **1** (Click Chemistry Tools, Scottsdale, USA), Ac_4GlcNAlk **2** (Click Chemistry Tools) or DMSO vehicle. After 7 h (cmpd. **5**) or 14 h (Ac_4GalNAlk, Ac_4GlcNAlk), cells were harvested, centrifuged at 500 g, 5 min, 4 °C and resuspended in PBS (1 mL). Cell pellets were resuspended in PBS (1 mL). 0.9 mL cell suspension transferred to O-ring tubes (1.5 mL, Thermo Fisher) and harvested. Zirconia/silica beads (0.1 mm, BioSpec, Bertlesville, USA) were added at a volume similar to the cell pellet, followed by 1:1 acetonitrile/water (1 mL). Cells were lysed using a bead beater (FastPrep-24, MP Biomedicals, Santa Ana, USA) at 6 m/s for 30 s, and the cell lysate was cooled at 4 °C for 10 min. Samples were centrifuged (14000 g, 10 min, 4 °C), and the supernatant was transferred to a fresh tube. The solvent was evaporated by speed vac, and the residue was dissolved in LCMS-grade water (Thermo Fisher, 0.2-0.4 mL) containing 15 µM ADP-α-D-glucose (Sigma-Aldrich, St. Louise, USA). The solution was passed through a centrifuge filter (30 min, 14000 g) using a 3 kDa MWCO Amicon Ultra Centrifugal Filter Unit (Merck, Kenilworth, USA). High performance anion exchange chromatography was used to analyze lysates. The residual cell suspension in PBS (0.1 mL) was used to confirm expression of AGX1 and GALE as reported recently.[1]

High performance anion exchange chromatography was carried out using an ICS-5000 with a quaternary pump and pulsed amperometric detection on a CarboPac PA1 4x250 mm column and a 4x50 mm guard column (Thermo Fisher). Solvents were: A = 1 mM NaOH in degassed water; B = 1 mM NaOH, 1M NaOAc

in degassed water. 0 min 10% B; 3 min 25% B; 8 min 28% B; 10 min 70% B; 23 min 75% B; 24 min 100% B; 27 min 100% B; 29 min 10% B; 32 min 10% B. Commercial or synthetic standards (200-500 µM) were used as controls.

Metabolic cell surface labeling, flow cytometry and in-gel fluorescence

K-562 cells stably transfected with pSBtet or pSBbi-based plasmids were seeded at a density of 250,000 cells/mL into well plates in growth medium without hygromycin. Cells were treated with DMSO, caged GalNAlk-1-phosphate analog **5**, Ac_4GalNAlk **1**, Ac_4ManNAlk, or Ac_4GlcNAlk **2** at the indicated concentrations and using either GalNAc or GlcNAc as additives in the indicated concentrations. Cells were grown for 20 h.

For sialidase treatment, an aliquot of cells (500 µL from a 1.5 mL suspension) were harvested, washed with serum-free RPMI media and treated with 40 U SialEXO (Genovis, Lund, Sweden) in 100 µL serum-free media for 3 h at 37 °C.

For in-gel fluorescence, cells were harvested in a V-shaped 96 well plate and washed twice with 2% FBS in PBS (Labeling Buffer, 0.2 mL). Cells were resuspended in Labeling Buffer (35 µL), treated with a solution of 100 µM $CuSO_4$, 600 µM BTTAA (Click Chemistry Tools), 2.5 mM sodium ascorbate, 2.5 mM aminoguanidinium chloride and 100 µM CF680 picolyl azide (Biotium, Fremont, USA) in Labeling Buffer (35 µL), and incubated for 7 min at room temperature on an orbital shaker. The click reaction was quenched with 3 mM bathocuproinedisulfonic acid (BCS) in PBS (35 µL). Cells were centrifuged, washed twice with Labeling Buffer and then with PBS, and treated with ice-cold Lysis Buffer (50 mM Tris-HCl pH 8, 150 mM NaCl, 1% (v/v) Triton X-100, 0.5% (v/v) sodium deoxycholate, 0.1% (w/v) SDS, 1 mM $MgCl_2$, and 100 mU/µL benzonase (Merck) containing cOmplete protease inhibitors, (0.1 mL). Cells were lysed for 20 min at 4 °C on an orbital shaker and centrifuged (1500 g, 20 min, 4 °C). Supernatant was transferred to a new plate and BCA was used to measure protein concentration. Loading buffer (a 1:1:1:0.5 (v/v/v/v) mixture of 1 M Tris-HCl pH 6.5, 80% (v/v) glycerol, 10% (w/v) SDS and 1 M DTT) was added, samples were heated at 95 °C for 30 s, run on a 10% or 4-20% Criterion™ gel (Bio-Rad, Hercules, USA) for SDS-PAGE, and imaged on an Odyssey CLx imager (LI-COR Biosciences, Lincoln, USA). Total protein was stained with Coomassie using Acquastain (Bulldog Bio, Portsmouth, USA). Protein expression was assessed by Western blot with a different set of samples, using antibodies against GALE (sc-390407, Santa Cruz Biotechnology, Dallas, USA), GAPDH (ab128915, abcam, Cambridge, UK), FLAG tag (mouse anti-FLAG M2, Sigma-Aldrich) or AGX1 (PA5-53206 Invitrogen, Carlsbad, USA).

For glycoprotease treatment, 1 million K-562 cells stably transfected with pSBbi-AGX1^{F383A} in 4 mL growth media were fed with 50 µM caged GalNAlk-1-phosphate **5** in Labeling Buffer (200 µL) as described above. Following washing, cells were suspended in Hank's Buffered Salt Solution (HBSS, 700 µL) and distributed into seven wells of a V-shaped 96 well plate. Cells were harvested and resuspended in HBSS containing StcE[5] or OpeRATOR/SialEXO (Genovis, Lund, Sweden) at the indicated concentrations. After incubation for 5 min at 37 °C, cells were harvested and supernatants were transferred into fresh tubes. Supernatants were treated with 10% (w/v) Rapigest in water, shock-frozen on dry ice, lyophilized and resuspended in 25% (v/v) Loading Buffer in water (25 µL). Cells were lysed in Lysis Buffer (40 µL) and in-gel fluorescence was performed as described above.

For flow cytometry, cells were harvested, washed twice with Labeling Buffer, resuspended in Labeling Buffer (50 µL), and treated with a solution of 100 µM $CuSO_4$, 600 µM BTTAA (Click Chemistry Tools),

2.5 mM sodium ascorbate, 2.5 mM aminoguanidinium chloride and 100 μM biotin picolyl azide (Click Chemistry Tools) in Labeling Buffer (50 μL). Cells were incubated for 7 min at room temperature on an orbital shaker, quenched with 3 mM BCS in PBS (50 μL) and washed twice with Labeling Buffer. Cells were treated with Dichlorotriazinylaminofluorescein (DTAF)-streptavidin (1:000, Jackson ImmunoResearch, Cambridge, UK), or mouse anti-biotin-Alexa Fluor 647 (1:200, Jackson ImmunoResearch) when assessing labeling of pSBtet-GH-transfected cells that express green fluorescent protein. Cells were incubated for 1 h at room temperature, washed twice and treated with SYTOX red (only DTAF-streptavidin-treated cells; 1:1000, Thermo Fisher) in Labeling Buffer. Flow cytometry was performed on an Accuri C6 flow cytometer (Becton Dickinson, Franklin Lakes, USA). Signal was gated for living cells either by SYTOX signal or forward and side scattering.

Fluorescence microscopy

Non-transfected and stably transfected 4T1 cells with pSBbi-AGX1 ^{F383A} were seeded into μ-Plate 24 Well Black (Thistle Scientific Ltd, Glasgow, UK) at a density of 30,000 cells in 350 μL growth medium without hygromycin. Cells were treated with either DMSO, 25 μM Ac_4GalNAlk or 10 μM Ac_4ManAlk. Cells were incubated for 16 h. Medium was aspirated, and cells were washed with ice-cold 2% (v/v) FBS in PBS (2x200 μL). Cells were then treated with 200 μL of a freshly prepared solution containing 300 μM BTTAA, 50 μM CuSO4, 5 mM sodium ascorbate, 5 mM aminoguanidinium chloride and 200 μM biotin-picolyl-azide (Click Chemistry Tools). The reaction was carried out for 3 min at room temperature, the supernatant was removed and cells were washed with ice-cold PBS (4x200 μL). Cells were incubated 20 min at RT with 20 μg/mL Streptavidin-AlexaFluor647 (BioLegend Ltd, Kentish Town, UK) in the dark. After washing with ice-cold PBS (4x200 μL), cells were fixed 20 min with cold 4% (v/v) formaldehyde (Thermo Fisher) in 100 mM sodium phosphate buffer pH 7.4, at room temperature in the dark. The reaction was quenched by 5 min incubation with 50 mM ammonium chloride and the cells were washed with PBS (3x200 μL). Cells were permeabilized with 0.1% (v/v) Triton X-100 in PBS for 10 min at 4 °C, and washed with PBS (3x200 μL). Cells were blocked in a solution of 10% (v/v) normal donkey serum (abcam), 1% (w/v) BSA and 0.1% (v/v) Tween-20 in PBS for 1 h at room temperature. Cells were incubated with DAPI (1:1000 v/v, Vector Laboratories Ltd, Peterborough, UK) in 1% (w/v) BSA and 0.1% (v/v) Tween-20 in PBS (Thermo Fisher) for 30 min at room temperature. After washing with PBS (3x200 μL), circle CoverSlips with 15 mm diameter (Thermo Fisher) were mounted onto each well by using ProLong Gold antifade reagent (Invitrogen).
The confocal acquisition was made on a Zeiss LSM710 Invert microscope. The images were acquired using a Plan Apochromat 20X/0.8 objective, with an acquisition zoom, so the resulting pixel size was 0.13 μm. A sequential scan to spectrally separate the fluorescence of DAPI and Alexa Fluor 647 was used. In addition, the transmitted light channel was activated to visualize the cell morphology. Images were visualized with Fiji software.[6]

Synthetic chemistry

Solvents and reagents were of commercial grade. Anhydrous solvents were obtained from a Dry Solvent System. Water-sensitive reactions were carried out in heat-dried glassware and under a nitrogen atmosphere. Thin layer chromatography (TLC) was performed on Kieselgel 60 F254 glass plates pre-coated with silica gel (0.25 mm thickness). Spots were developed with ceric ammonium molybdate stain (5% (w/v) ammonium molybdate, 1% (w/v) cerium (II) sulfate and 10% (v/v) sulfuric acid in water) or sugar stain (0.1% (v/v) 3-methoxyphenol, 2.5% (v/v) sulfuric acid in EtOH) dipping solutions. Flash chromatography

was carried out on Fluka Kieselgel 60 (230-400 mesh). Solvents were removed under reduced pressure using a rotary evaporator and high vacuum (1 mbar).

^{1}H, ^{13}C and 2D NMR spectra were measured with an AS400 spectrometer, an AS600 spectrometer (Varian, Palo Alto, USA) or a Bruker Avance-400 MHz spectrometer at 298 K. Chemical shifts (σ) are reported in parts per million (ppm) relative to the respective residual solvent peaks ($CDCl_3$: σ 7.26 in ^{1}H and 77.16 in ^{13}C NMR; acetone-D_6: σ 2.05 in ^{1}H and 29.84 in ^{13}C NMR; D_2O: σ 4.79 in ^{1}H). Two-dimensional NMR experiments (HH-COSY, CH-HSQC) were performed to assign peaks in ^{1}H spectra. The following abbreviations are used to indicate peak multiplicities: s singlet; d doublet; dd doublet of doublets; dt doublet of triplets; m multiplet. Coupling constants (*J*) are reported in Hertz (Hz). High resolution mass spectrometry by electrospray ionization (ESI-HRMS) was performed at Stanford University Mass Spectrometry, with a micrOTOF-Q II hybrid quadrupole time-of-flight mass spectrometer (Bruker, Billerica, USA) equipped with a 1260 UPLC (Agilent). Low resolution mass spectrometry by electrospray ionization (ESI-LRMS) was performed on an UPLC-MS (Waters) equipped with ACQUITY UPLC® BEH C18 column.

UDP-GalNAlk[2], UDP-GlcNAlk[7] and caged GalNAzMe-1-phosphate **8**[1] have been made previously. Ac_4GlcNAlk, Ac_4GalNAlk, Ac_4ManNAlk, Ac_4GlcNAz and Ac_4GalNAz are commercially available (Click Chemistry Tools).

Bis(*S*-acetyl-2-thioethyl) 3,4,6-tri-*O*-acetyl-2-deoxy-2-(4-pentynoyl)amido-α-D-galactopyranosyl phosphate (5)

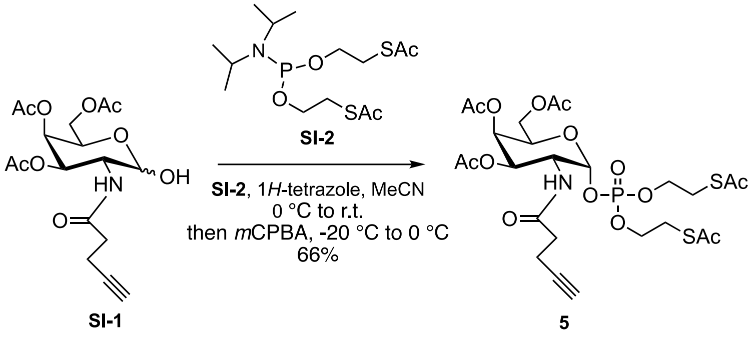

To a stirred solution of lactol **SI-1** (100 mg, 259 µmol) in MeCN (1 mL) were added at 0 °C 1*H*-tetrazole (27.1 mg, 388 µmol, 873 µL of a 3% (w/v) solution in MeCN) and phosphoramidite **SI-2**[8] (133 mg, 362 µmol) in 5:1 MeCN/toluene (1.2 mL). The reaction was warmed to room temperature and stirred for 1 h. The mixture was cooled to -20 °C and treated with mCPBA (66.9 mg, 388 µmol). The mixture was warmed to 0 °C, stirred for 20 min at that temperature, and treated with mCPBA (22 mg, 128 µmol) to drive the reaction to completion. The reaction was stirred for 20 min and quenched with 10% aq. Na_2SO_3 (10 mL). The solution was extracted with DCM (5x10 mL), the combined organic layers were dried over $MgSO_4$, filtered and concentrated. The residue was purified by flash chromatography (hexanes/EtOAc 1:0 to 2:3 with 0.5% (v/v) NEt_3) to give phosphotriester **5** (115 mg, 171 µmol, 66%) as a clear oil. Rf (hexanes/EtOAc 2:3 with 0.5% (v/v) NEt_3, TLC plates pre-neutralized with hexanes/2% NEt_3) = 0.4. ^{1}H NMR (600 MHz, $CDCl_3$) δ 6.27 (d, *J* = 9.2 Hz, 1H), 5.77 (dd, *J* = 5.5, 3.3 Hz, 1H), 5.45 (dd, *J* = 3.3, 1.3 Hz, 1H), 5.20 (dd, *J* = 11.5, 3.2 Hz, 1H), 4.73 (ddt, *J* = 12.3, 9.4, 3.3 Hz, 1H), 4.45 – 4.37 (m, 1H), 4.27 – 4.04 (m, 6H), 3.31 – 3.09 (m, 4H), 2.57 – 2.40 (m, 3H), 2.38 (s, 2H), 2.37 (s, 4H), 2.16 (s, 2H), 2.04 (s, 2H), 2.02 – 1.97 (m, 3H). ^{13}C NMR (150 MHz, $CDCl_3$) δ 195.3, 194.9, 171.6, 170.7, 170.4, 170.2, 97.3, 82.9, 69.5, 69.3, 68.8, 67.4, 67.2, 66.9, 66.8, 66.6, 66.5, 66.5, 61.4, 47.6, 47.5, 47.4, 35.1, 30.8, 30.6, 29.2, 29.2, 20.8, 20.8, 20.7, 14.8; HRMS (ESI) calcd. for $C_{25}H_{36}NO_{14}PS2$ (M+Na+) 692.1213 found 692.1204 *m/z*.

2-deoxy-2-(4-pentynoyl)amido-α-D-galactopyranosyl phosphate disodium salt (SI-5)

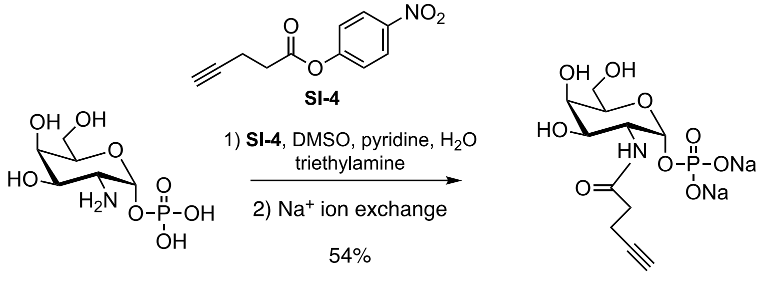

Amine **SI-3** (15 mg, 58 µmol) in anhydrous DMSO/pyridine (1:1.5 v/v, 1.2 mL) was treated with 4-nitrophenyl pent-4-ynoate[9] (19 mg, 87 µmol) and triethylamine (8 µL, 58 µmol). The turbid solution was left to stir overnight. The reaction was treated with 100 µL water and left to stir for another 8 h. Another 150 µL water were added as well as 1.5 equiv. reagent **SI-4** (19 mg, 87 µmol). After another 60 h, another 200 µL water were added as well as, then another 1.5 equiv. reagent **SI-4** (19 mg, 87 µmol). The yellow solution turned clear overnight, when TLC (DCM/MeOH/water 4:1:1 with 2 drops triethylamine) indicated conversion. The solution was shock frozen on dry ice, lyophilized and purified by size exclusion chromatography (Sephadex G-25 extra fine, Sigma-Aldrich) using water/MeOH 3:2 (v/v) as a solvent. The combined fractions were concentrated and passed through AG 50W-X8 Na^+ form resin (Bio-Rad) and lyophilized to give alkyne **SI-5** (12 mg, 31 µmol, 54%) as a white solid. ^{1}H NMR (600 MHz, D_2O) δ 5.50 – 5.45 (m, 1H), 4.29 – 4.22 (m, 1H), 4.17 (s, 1H), 4.05 (d, *J* = 3.4 Hz, 1H), 3.97 (s, 1H), 3.82 – 3.73 (m, 2H), 2.60 – 2.49 (m, 4H), 2.41 – 2.36 (m, 1H); ^{13}C NMR (150 MHz, D_2O) δ 175.5, 132.7, 93.8, 72.0, 71.8, 70.5, 69.9, 68.7, 68.6, 67.6, 67.5, 61.3, 50.2, 34.5, 14.5. HRMS (ESI) calcd. for $C_{11}H_{18}NO_9P$ (M-H^+) 338.0646 found 338.0648 *m/z*.

Characterization of synthetic compounds

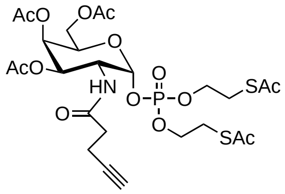

^{1}H NMR (600 MHz, $CDCl_3$)

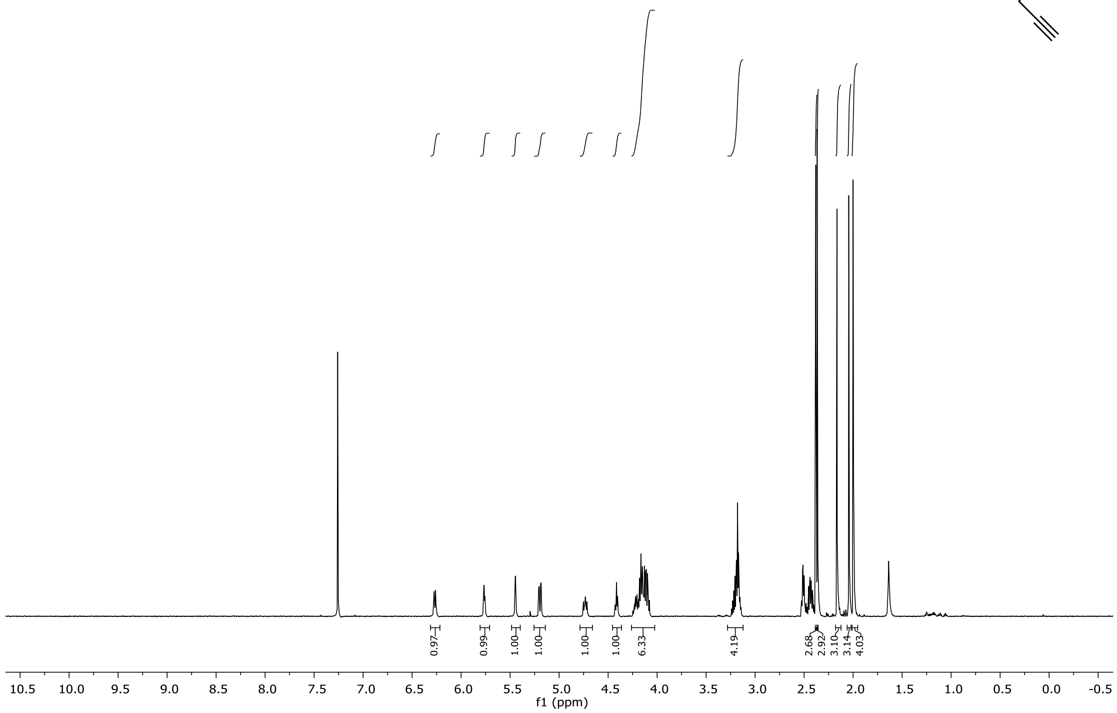

^{13}C NMR (150 MHz, $CDCl_3$)

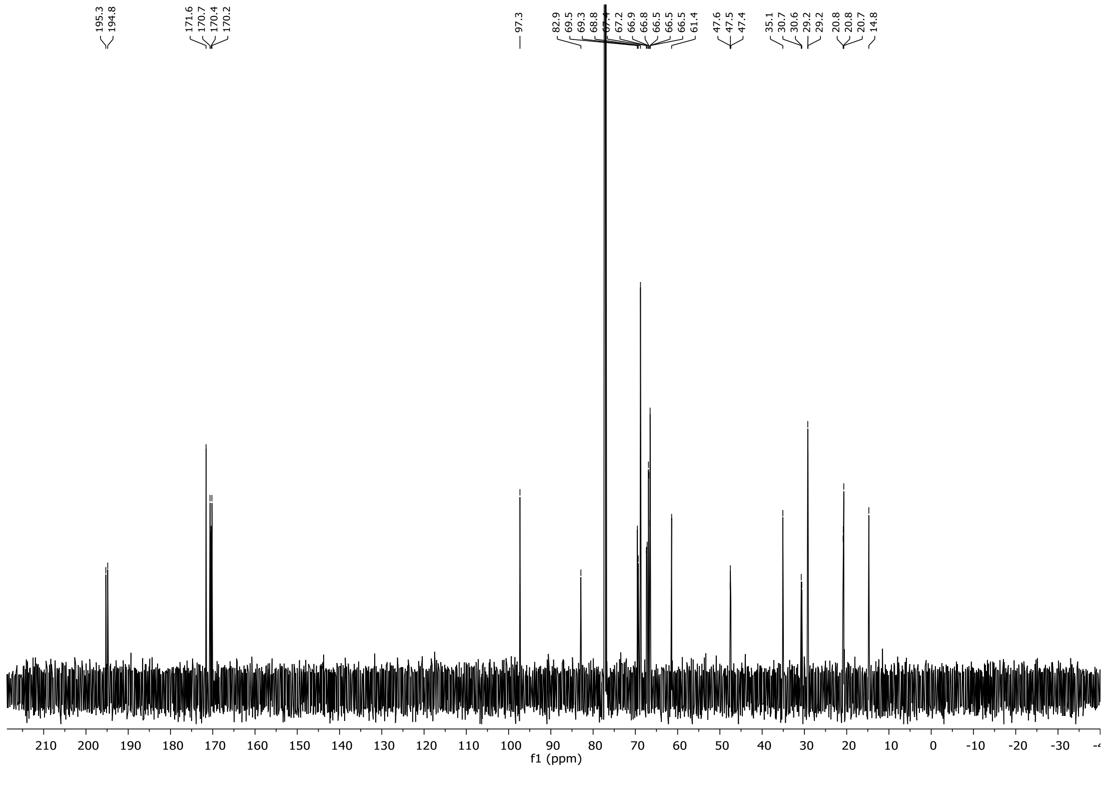

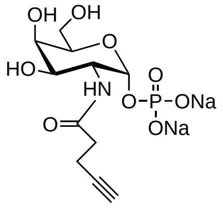

^{1}H NMR (600 MHz, D_2O)

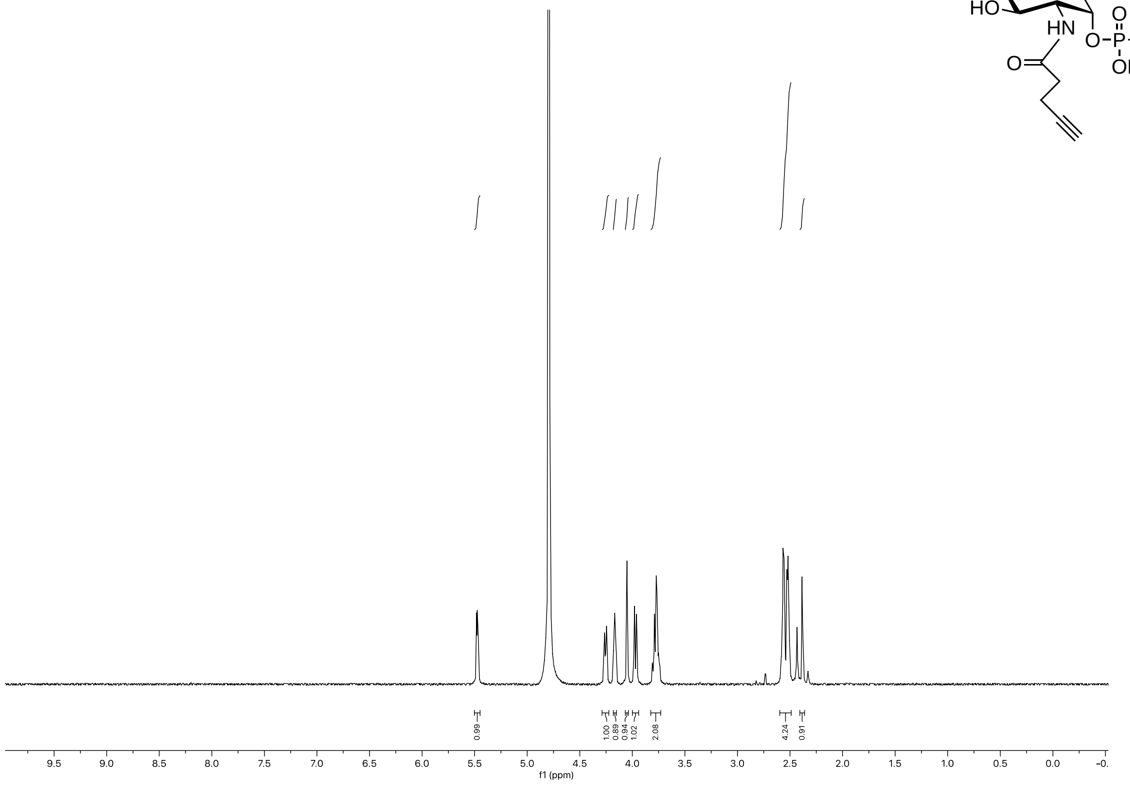

^{13}C NMR (150 MHz, D_2O)

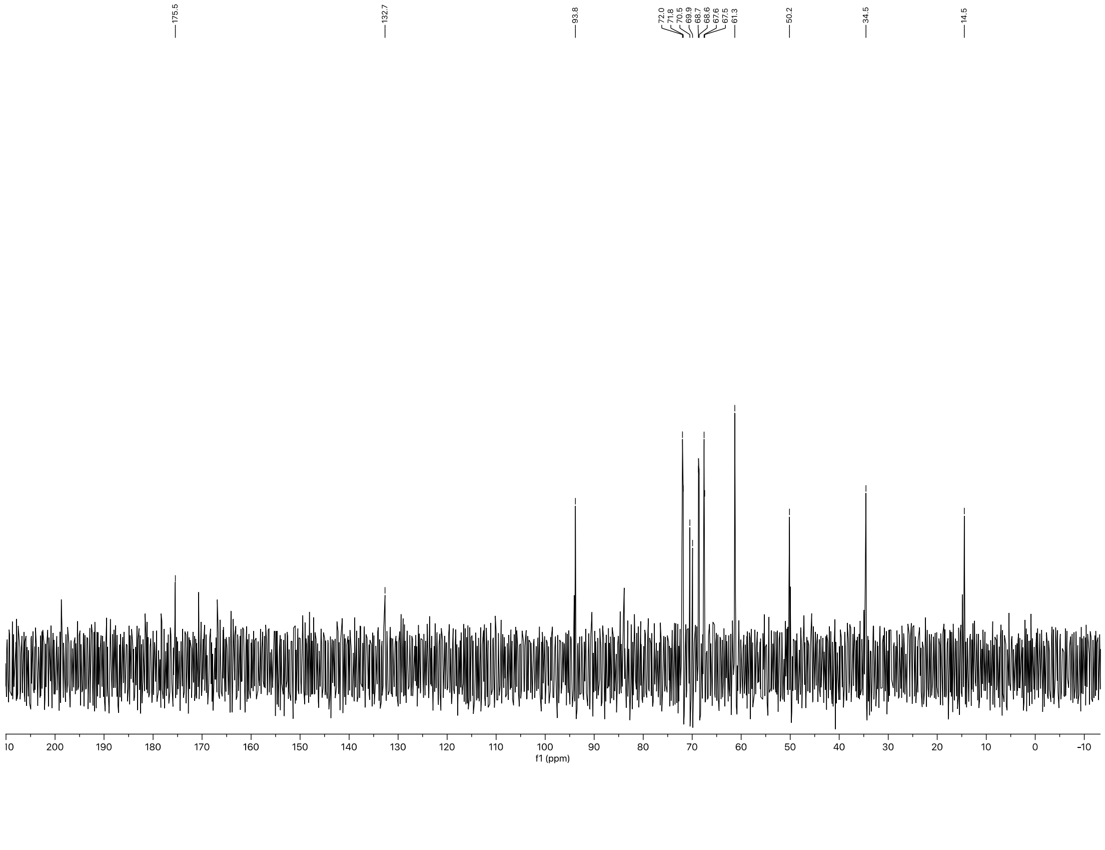

References

(1) Debets, M. F.; Tastan, O. Y.; Wisnovsky, S. P.; Malaker, S. A.; Angelis, N.; Moeckl, L. K. R.; Choi, J.; Flynn, H.; Wagner, L. J. S.; Bineva-Todd, G.; Antonopoulos, A.; Cioce, A.; Browne, W. M.; Li, Z.; Briggs, D. C.; Douglas, H. L.; Hess, G. T.; Agbay, A. J.; Roustan, C.; Kjaer, S.; Haslam, S. M.; Snijders, A. P.; Bassik, M. C.; Moerner, W. E.; Li, V. S. W.; Bertozzi, C. R.; Schumann, B. Metabolic Precision Labeling Enables Selective Probing of O-Linked N -Acetylgalactosamine Glycosylation . *Proc. Natl. Acad. Sci. U. S. A.* **2020**, *117* (41), 25293–25301. https://doi.org/10.1073/pnas.2007297117.

(2) Choi, J.; Wagner, L. J. S.; Timmermans, S. B. P. E.; Malaker, S. A.; Schumann, B.; Gray, M. A.; Debets, M. F.; Takashima, M.; Gehring, J.; Bertozzi, C. R. Engineering Orthogonal Polypeptide GalNAc-Transferase and UDP- Sugar Pairs. **2019**. https://doi.org/10.1021/jacs.9b04695.

(3) Schumann, B.; Malaker, S. A.; Wisnovsky, S. P.; Debets, M. F.; Agbay, A. J.; Fernandez, D.; Wagner, L. J. S.; Lin, L.; Li, Z.; Choi, J.; Fox, D. M.; Peh, J.; Gray, M. A.; Pedram, K.; Kohler, J. J.; Mrksich, M.; Bertozzi, C. R. Bump-and-Hole Engineering Identifies Specific Substrates of Glycosyltransferases in Living Cells. *Mol. Cell* **2020**, *78*, 1–11. https://doi.org/10.1016/j.molcel.2020.03.030.

(4) Kowarz, E.; Löscher, D.; Marschalek, R. Optimized Sleeping Beauty Transposons Rapidly Generate Stable Transgenic Cell Lines. *Biotechnol. J.* **2015**, *10* (4), 647–653. https://doi.org/10.1002/biot.201400821.

(5) Malaker, S. A.; Pedram, K.; Ferracane, M. J.; Bensing, B. A.; Krishnan, V.; Pett, C.; Yu, J.; Woods, E. C.; Kramer, J. R.; Westerlind, U.; Dorigo, O.; Bertozzi, C. R. The Mucin-Selective Protease StcE Enables Molecular and Functional Analysis of Human Cancer-Associated Mucins. *Proc. Natl. Acad. Sci. U. S. A.* **2019**, *116* (15), 7278–7287. https://doi.org/10.1073/pnas.1813020116.

(6) Schindelin, J.; Arganda-Carreras, I.; Frise, E.; Kaynig, V.; Longair, M.; Pietzsch, T.; Preibisch, S.; Rueden, C.; Saalfeld, S.; Schmid, B.; Tinevez, J. Y.; White, D. J.; Hartenstein, V.; Eliceiri, K.; Tomancak, P.; Cardona, A. Fiji: An Open-Source Platform for Biological-Image Analysis. *Nat. Methods* **2012**, *9* (7), 676–682. https://doi.org/10.1038/nmeth.2019.

(7) Wen, L.; Gadi, M. R.; Zheng, Y.; Gibbons, C.; Kondengaden, S. M.; Zhang, J.; Wang, P. G. Chemoenzymatic Synthesis of Unnatural Nucleotide Sugars for Enzymatic Bioorthogonal Labeling. *ACS Catal.* **2018**, *8* (8), 7659–7666. https://doi.org/10.1021/acscatal.8b02081.

(8) Yu, S. H.; Boyce, M.; Wands, A. M.; Bond, M. R.; Bertozzi, C. R.; Kohler, J. J. Metabolic Labeling Enables Selective Photocrosslinking of O-GlcNAc-Modified Proteins to Their Binding Partners. *Proc. Natl. Acad. Sci. U. S. A.* **2012**, *109* (13), 4834–4839. https://doi.org/10.1073/pnas.1114356109.

(9) Punna, S.; Kaltgrad, E.; Finn, M. G. “Clickable” Agarose for Affinity Chromatography. *Bioconjug. Chem.* **2005**, *16* (6), 1536–1541. https://doi.org/10.1021/bc0501496.
